# Directing cellular responses in a nanocomposite 3D matrix for tissue regeneration with nanoparticle-mediated drug delivery

**DOI:** 10.1016/j.mtbio.2023.100865

**Published:** 2023-11-14

**Authors:** Ezgi Özliseli, Sami Şanlıdağ, Behice Süren, Alaa Mahran, Marjaana Parikainen, Cecilia Sahlgren, Jessica M. Rosenholm

**Affiliations:** a Pharmaceutical Sciences Laboratory, Faculty of Science and Engineering, Åbo Akademi University, Turku, Finland; b Faculty of Science and Engineering, Biosciences, Åbo Akademi University, Turku, Finland; c InFLAMES Research Flagship Center, Åbo Akademi University, Turku, Finland; d Turku Bioscience Centre, Åbo Akademi University and University of Turku, Turku, Finland; e Department of Pharmaceutics, Faculty of Pharmacy, Assiut University, Assiut, 71526, Egypt; f Department of Biomedical Engineering, Eindhoven University of Technology, Eindhoven, the Netherlands; g Institute for Complex Molecular Systems (ICMS), Eindhoven University of Technology, Eindhoven, the Netherlands

**Keywords:** Mesoporous silica nanoparticles, Nanocomposite hydrogel, Surface modification, Hydrophobic drug delivery, 3D cell culture, Tissue engineering

## Abstract

Hydrogels play an important role in tissue engineering due to their native extracellular matrix-like characteristics, but they are insufficient in providing the necessary stimuli to support tissue formation. Efforts to integrate bioactive cues directly into hydrogels are hindered by incompatibility with hydrophobic drugs, issues of burst/uncontrolled release, and rapid degradation of the bioactive molecules. Skeletal muscle tissue repair requires internal stimuli and communication between cells for regeneration, and nanocomposite systems offer to improve the therapeutic effects in tissue regeneration. Here, the versatility of mesoporous silica nanoparticles (MSN) was leveraged to formulate a nanoparticle-hydrogel composite and to combine the benefits of controlled delivery of bioactive cues and cellular support. The tunable surface characteristics of MSNs were exploited to optimize homogeneity and intracellular drug delivery in a 3D matrix. Nanocomposite hydrogels formulated with acetylated or succinylated MSNs achieved high homogeneity in 3D distribution, with succinylated MSNs being rapidly internalized and acetylated MSNs exhibiting slower cellular uptake. MSN-hydrogel nanocomposites simultaneously allowed efficient local intracellular delivery of a hydrophobic model drug. To further study the efficiency of directing cell response, a Notch signaling inhibitor (DAPT) was incorporated into succinylated MSNs and incorporated into the hydrogel. MSN-hydrogel nanocomposites effectively downregulated the Notch signaling target genes, and accelerated and maintained the expression of myogenic markers. The current findings demonstrate a proof-of-concept in effective surface engineering strategies for MSN-based nanocomposites, suited for hydrophobic drug delivery in tissue regeneration with guided cues.

## Graphical abstract

**Figure:**
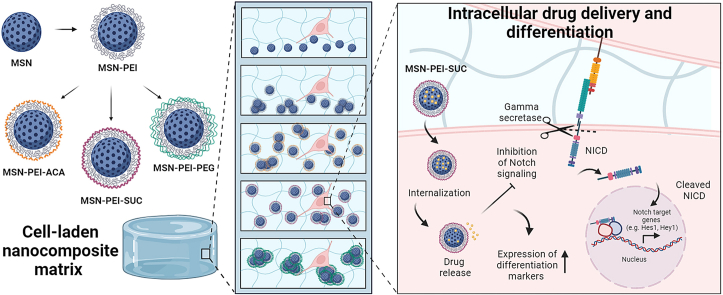

## 1 Introduction

In the past decades, tissue engineering (TE) has advanced as an interdisciplinary field to provide new solutions for tissue and organ regeneration, and it is among the most promising strategies for replacing damaged or lost tissue such as heart, neuronal, vascular, and muscle tissues [1,2]. TE practices mainly employ patient-derived stem cells to construct new tissue by using biomaterials and biological cues, thereby avoiding immune rejection [3]. However, challenges remain in terms of developing functional biomaterials that mechanically and biologically promote cell attachment, subsequent proliferation, and differentiation by providing the necessary biochemical cues [4,5].

The extracellular matrix (ECM) that surrounds cells in natural tissues presents chemical and physical signals to regulate tissue repair and contributes to several cell dynamics including cell adhesion, migration, proliferation, and differentiation [6]. Hydrogels have been widely utilized as ECM analogs due to their hydrophilic nature to provide a supportive environment for cells to grow and differentiate into functional tissue. A wide variety of natural and synthetic polymers, as well as their combinations, have been extensively investigated for native soft tissue such as skin, muscle, fat, or nerve in tissue engineering [7,8]. Among them, natural polymer collagen type I, the most abundant protein in the majority of mammalian tissue extracellular matrices, is widely studied for two-dimensional (2D) and three-dimensional (3D) cell culture and TE due to its physiological relevance and having inherent features such as biocompatibility, molecular composition, architecture and biodegradability [5,9,10]. In addition to the strategies for developing new biomaterials mimicking the native ECM with suitable surface chemistry, topography, and porosity, new methods for incorporating biochemical cues into hydrogels to direct and coordinate cellular activities have been the major focus for increasing the success of tissue regeneration and repair [8].

Non-covalent and covalent binding of bioactive cues onto hydrogels are primarily utilized for the delivery of hydrophilic compounds such as peptides, proteins, and hydrophilic drugs, but their effectiveness in delivering hydrophobic drugs is hindered by low loading quantity and lack of homogeneity in the hydrogel matrices [11,12]. Considering small-molecule drugs are the most patented in the market [13], about 90% of preclinical drug candidates are compounds with poor water solubility [14], and several different small-molecule drugs are being developed to promote stem cell proliferation or differentiation [15]; novel approaches have been investigated for incorporating these hydrophobic compounds into hydrogels. For direct attachment of drug molecules to the matrix, approaches such as incorporating hydrophobic moieties to alter surface modification and grafting inclusion complexes (e.g. cyclodextrins) onto polymer structures have been developed [16]. However, these attempts have proved insufficient in achieving high drug loading, uniform drug distribution, preventing drug recrystallization, and controlling drug release as well as causing biosafety concerns [11,17].

Nanoparticles can act as a reservoir of drug molecules in hydrogels while offering distinguished benefits, such as 1) reduction of initial burst release, followed by 2) sustained or even controlled release, 3) protection of the encapsulated biological molecules against degradation and premature clearance and elimination, and 4) enhanced solubility of hydrophobic drugs at the site of action [[18], [19], [20]]. Among the vast types of nanomaterials developed, silica-based materials, and especially mesoporous silica nanoparticles (MSNs) have come forward as highly flexible nanomedical drug delivery platform owing to their biocompatibility, high specific surface area, and tunable particle and pore size to fit desired cargo molecules, as well as high flexibility for surface engineering [[21], [22], [23]]. Moreover, imaging moieties can readily be incorporated into MSNs during the synthesis process to monitor and guide the therapeutic intervention on a real-time basis [24]. New generation heterosilica-based nanoparticles have gained substantial attention in recent years in drug delivery and photon-based therapies, exhibiting promising results in biomedicine. These advancements involve the integration of MSNs with other nanoparticle types, leading to the creation of multifunctional MSN-based hybrid drug delivery systems such as Janus nanoparticles and nanotrucks [[25], [26], [27]]. Surface modification represents one of the main strategies to achieve desired properties for developing nano-delivery platforms which can facilitate improved biocompatibility, colloidal stability, as well as the rate and mechanism of cellular uptake [24]. Additionally, surface modification can be utilized to improve drug-MSN interactions along with high drug loading efficiency, control the drug release functions, and provide control over diffusional transport to achieve controlled drug release [[28], [29], [30]].

In tissue engineering, one promising strategy is to develop hybrid nanoparticle-hydrogel composite systems for local and controlled release of bioactive moieties for improved regeneration [31]. These nanocomposite hydrogels can act as a cellular substrate with advanced properties combining the benefit of hydrogels, which provide an architectural host environment and reservoir for nutrients for cells, and nanoparticles that act as vehicles for mediated release of bioactive molecules, thereby promoting stem cell attachment and directing the stem cell fate *in situ* [3,32]. Although MSNs have been widely studied in cancer treatment and other therapeutic settings, the use of MSNs in regenerative medicine is a relatively new concept, and hydrogel-MSN-cell interactions require further investigation. Several studies have shown efficient drug delivery using silica-based nanocomposite hydrogels from both 2D surfaces and 3D nanocomposites with natural polymers (e.g. gelatin methacrylate, hyaluronic acid, alginate/gelatin) and synthetic polymers. These studies demonstrated that silica-based nanocomposites have a wide range of potential for antibacterial delivery [33], vasculogenesis [34], wound healing [35], cancer treatment [36], pulpitis therapy [37], and bone tissue engineering [[38], [39]]. However, the majority of the material compatibility and functionality studies were conducted in 2D cell monolayers, which do not closely recapitulate *in vivo* conditions [40]. Animal studies are better suited for demonstrating the overall functionality, but involve the impact of various factors on the entire organism, making it challenging to study basic biological mechanisms and material-cell interactions [41].

Hereby, we developed MSN-collagen composite (nanocomposite) hydrogels for tissue engineering that facilitate intracellular drug delivery. Our study aimed to utilize collagen, a natural material with proven biocompatibility, given the versatility of FDA-approved collagen-based nanomaterials [[42], [43], [44]], and provide a systematical understanding of the impact of surface modification of MSNs in 3D culture for the formulation of MSN-collagen nanocomposite hydrogels that can be utilized for tissue regeneration. We modified the MSN surfaces with polyethylene imine (PEI) polymer, known to improve cellular internalization, and further derivatized the surface via acetylation (ACA), succinylation (SUC), or polyethylene glycol polymer (PEG) conjugation. We evaluated the influence of MSN surface modifications on the homogeneity of the formulation as a nanocomposite hydrogel. We observed improved biocompatibility, enhanced cellular internalization of cargo, and controlled and localized hydrophobic drug delivery within the 3D matrix. Our results emphasize the importance of surface charge in relation to the matrix-nanoparticle interactions when designing MSN-hydrogel-based composite systems.

Finally, we employed a gamma-secretase inhibitor (GSI), (2*S*)–*N*-[(3,5-Difluorophenyl)acetyl]-L-alanyl-2-phenyl]glycine 1,1-dimethyl ethyl ester, also known as DAPT, as a proof-of-concept to study the ability of the MSNs to deliver molecular cargo within the nanocomposite hydrogel. DAPT inhibits Notch signaling, an important regulator of cell fate during tissue development and regeneration, by blocking the gamma-secretase-mediated Notch receptor cleavage resulting in downregulation of the expression of several known target genes (e.g., Hes1 and Hey1) [45]. We have shown that MSNs loaded with DAPT in the nanocomposite hydrogel were able to downregulate the expression of the transcriptional targets of Notch signaling in C2C12 cells and induce the early and late myogenic markers. Our findings demonstrate that the nanocomposite hydrogel can be successfully used to deliver molecular cargo to control cell behavior. Due to its multifunctionality, it can be adapted for a wide range of tissue engineering applications in conjunction with other hydrophobic drug molecules.

## 2 Materials and methods

Unless otherwise stated, all chemicals were used as received, and Millipore water was used in the preparation of all aqueous solutions. Anhydrous ethanol (99.5%) was used for chemical reactions, purification, and storage of MSN. Cetyltrimethylammonium bromide (CTAB) and 1,3,5-trimethylbenzene (TMB, 99%) were purchased from ACROS. Decane (99%) was purchased from Alfa Aesar. Anhydrous toluene, anhydrous chloroform, ethylene glycol (99%), tetraethyl orthosilicate (TEOS) (99%), 3-aminopropyltriethoxysilane (APTES) (99%), ammonium hydroxide NH4OH (30wt%), ammonium nitrate, acetone, anhydrous cyclohexane, succinic anhydride, acetic anhydride, hexamethylene diisocyanate (HMDI), mPEG (methoxy polyethylene glycol, molecular weight 5000), tetramethylrhodamine isothiocyanate (TRITC), and collagenase from Clostridium histolyticum (C0130) type I were purchased from Sigma-Aldrich. Aziridine monomer was purchased from Menadiona. N-2-hydroxyethyl piperazine-N′-2-ethane sulfonic acid (HEPES) was purchased from R&D systems. Gamma secretase inhibitor DAPT (Cat. No. 2634) was purchased from TOCRIS and Alamar Blue (Resazurin cell proliferation assay) was purchased from TCI Europe. Acetic acid (100%) was purchased from J.T. Baker. 3,3′-Dioctadecyloxacarbocyanine perchlorate (DiO), Alexa Fluor Phalloidin 633 nm, CellMask™ Deep Red Plasma membrane stain, and DAPI were purchased from Thermo Fisher. Collagen type I rat tail was purchased from Corning.

### 2.1 MSN synthesis

MSNs were synthesized according to our previously published protocols by utilizing TEOS and APTES silica sources to achieve amino co-condensed mesoporous silica nanoparticles (MSN-NH2, abbreviated as MSN to simplify the starting nanoparticle) [46,47]. Briefly, a mixed solution was prepared by dissolving and heating CTAB (0.45 g) in a mixture of water (150 mL) and ethylene glycol (30 mL) at 70 °C in a flask reactor. Swelling agents decane and TMB were added to the mixture and allowed to emulsify for 2 h. Following, ammonium hydroxide (33 wt%, 2.5 mL) was introduced to the system as a catalyst before TEOS (1.5 mL) and APTES (0.3 mL) were added to initiate the reaction. Pre-treated APTES-TRITC complex [48] was added in the synthesis step with silica sources to create inherently fluorescent particles (T-MSNs) for fluorescence-assisted experiments. The reaction was allowed to proceed for 3 h at 70 °C. Then, the heating was stopped, and the as-synthesized colloidal suspension was then aged at 70 °C without stirring for 18 h. The molar composition of the synthesis solution was 1TEOS: 0.19APTES: 0.18CTAB: 0.55TMB: 1.6decane: 5.9NH3: 88.5ethylene glycol: 1249H2O [47] After the suspension was cooled to room temperature, nanoparticles were collected with acetone precipitation and surfactant template was removed by sonication in an ethanolic solution of ammonium nitrate (20 g/L) [49] four times followed by ethanol washing and stored as a suspension in anhydrous ethanol at 4 °C.

### 2.2 Surface modification of MSN

MSNs were further functionalized by surface hyperbranched polymerization of polyethyleneimine (PEI) as described by our previous works [46,50,51]. Briefly, MSN (100 mg) was dispersed in toluene (10 mL) and aziridine (52 μL) was added to the mixture in the presence of catalytic amounts of acetic acid (100%, 5.2 μL) and the reaction was allowed to proceed under stirring for 18 h at 35 °C. The resulting MSN-PEI nanoparticles were collected by centrifugation and washed with ethanol twice. Furthermore, to investigate the surface charge effect of nanoparticles, MSN-PEI were modified with succinic anhydride, acetic anhydride, or mPEG.

Carboxylated nanoparticles were achieved by suspending MSN-PEI (50 mg) in ethanol with the final concentration of 1 mg/mL and adding 10 mL of succinic anhydride solution (5 mg/mL in ethanol) under stirring. The suspension was stirred at room temperature for 18 h, separated by centrifugation, washed with ethanol three times, and prepared MSN-PEI-SUC nanoparticles were stored as a suspension in ethanol for further studies.

To achieve neutrally charged nanoparticles, primary amines in MSN-PEI were capped by uncharged acetyl groups in acetic anhydride [52]. This was accomplished by dispersing MSN-PEI (50 mg) in ethanol (50 mL) and adding acetic anhydride (0.4 mL) into the reaction while stirring. The reaction suspension was kept for 18 h and acetylated MSN was finally purified and resuspended in ethanol as for other modifications.

PEG-modified nanoparticles were prepared by first activating the mPEG with catalytic amounts of hexamethylene diisocyanate (HMDI) in chloroform. Once the mPEG is activated and dried, 200 wt% excess was added to a suspension of MSN-PEI in chloroform (1 mg/mL) and allowed to conjugate overnight at 60 °C under reflux. Finally, the suspension was washed two times and resuspended in ethanol [50].

### 2.3 Characterization of MSN

#### 2.3.1 Dynamic light scattering (DLS) and zeta-potential (ζ-potential) measurements

The hydrodynamic size and zeta potential of nanoparticles were characterized by using the Zetasizer Nano ZS instrument. DLS was carried out by dispersing nanoparticles in Milli-Q water with a final concentration of 0.1 mg/mL, and ζ-potential was investigated by dispersing nanoparticles in HEPES buffer (25 mM, pH 7.2) at fixed physiologically relevant pH [53]. Samples were sonicated and vortexed alternatively for 10 min and transferred to disposable cuvettes (DLS) or disposable folded capillaries (ζ-potential) for measurement. Each sample was measured three times and an average of hydrodynamic size, polydispersity index (PDI), and ζ-potential data with standard deviation was reported.

#### 2.3.2 Transmission electron microscopy

TEM grids were prepared by dropping MSN suspension (10 μL) with a concentration of 0.1 mg/mL in ethanol on a copper grid and kept for drying overnight. Images were acquired by JEM-1400 Plus Electron Microscope operating at 80 kV equipped with OSIS Quemesa 11 Mpix bottom-mounted digital camera. TEM was used to confirm the size, spherical morphology, and the formation of porous mesostructures.

#### 2.3.3 Thermogravimetric analysis (TGA)

Thermogravimetric analysis was conducted to further confirm the surface modification with the Netzsch STA 449F1 simultaneous TGA/DSC instrument. Nanoparticles were vacuum dried overnight and alumina crucibles containing approximately 2 mg sample were used for the analysis. Thermal analysis data was collected in the 25−900 °C range, under oxygen flow (heating rate = 10 K/min; flow rate = 30 mL/min) using argon as the carrier.

#### 2.3.4 Drug loading and quantification

Drug loading into MSN was realized via the adsorption method by using a non-polar solvent that strengthens the driving forces of the hydrophobic drug adsorption to the pore walls [54]. Prior to drug loading, nanoparticles were carefully vacuum dried and dispersed in cyclohexane at the concentration of 2 mg/mL using Covaris focused ultrasonicator. The desired amounts of dialkylcarbocyanine membrane probe (DiO) or (2*S*)–*N*-[(3,5-Difluorophenyl)acetyl]-L-alanyl-2-phenyl]glycine 1,1-dimethyl ethyl ester (DAPT) was directly added onto the suspension, the reaction mixture was ultrasonicated and agitated overnight at room temperature [22,55]. Drug-loaded nanoparticles were centrifuged and vacuum dried overnight to eliminate residual solvent and stored at 4ᵒC for further use. Confirmation of the drug loading was applied by elution of the known amount of loaded MSN into methanol (for DAPT) and DMSO (for DiO). Following 30 min of sonication, the supernatant was separated by centrifugation. The analysis was made for drug (DiO/DAPT) content by either UV–visible spectrophotometry (DiO) or HPLC (DAPT). The absorbance measurement was correlated with a calibration curve based on known concentration. (Analysis wavelength DiO = 484 nm, NanoDrop 2000c, Thermo Scientific, DAPT = 222 nm, Agilent 1100 series (Agilent Technologies, CA, USA)). DAPT quantification set-up consisted of a UV detector (diode array), Inertsil ODS-3 4.6 × 150 mm, and a 5 μm particle size column (GL Science). The flow rate was 1 mL/min, and the eluents were water and methanol. A linear gradient of 50% methanol to 100% methanol over 10 min was used. Calibration curves and chromatographic peaks received from HPLC are attached in the supplementary data.

### 2.4 Cell culture

C2C12 cell line was cultured in DMEM high glucose (Lonza) supplemented with 10% heat-inactivated FBS (Gibco), 2 mM l-glutamine, 100 units/mL penicillin, and 100 μg/mL streptomycin using standard conditions (37 °C, 5% CO_2_ and >95% humidity). Differentiation medium (DM) was prepared by replacing 10% FBS with 1% heat-inactivated horse serum.

### 2.5 3D nanocomposite matrix preparation

A 3D nanocomposite hydrogel (3D matrix) was prepared using collagen type I rat tail solution (4.07 mg/mL, LOT#0041007), according to the supplier's protocol with modifications to tailor for MSN incorporation. 10X PBS buffer was replaced with 10X HEPES buffered saline (pH 7.4), dH_2_0 was replaced with DMEM high glucose, and 1 M NaOH was replaced with 1 M KOH to protect the colloidal stability of nanoparticles within the matrix, as it is well established that phosphate ions form covalent-like bonds with amine groups [56], resulting in poor colloidal stability of surface modified MSNs. Briefly, collagen type I rat tail (491 μL) was reconstituted (as 1 mL final volume) and neutralized by adding 10X Hank's Balanced Salt solution (HBSS) (100 μL), DMEM (397.7 μL), and 1 M KOH (11.3 μL) to achieve 3D nanocomposite hydrogel with the final concentration of 2 mg/mL. Nanoparticles were incorporated from a stock solution in HEPES buffer and further homogenized with sonication with the specific order as follows: HBSS, 1 M KOH, DMEM, collagen, MSN. Cells were pipetted gently into MSN-incorporated neutralized gels to avoid cell stress and the prepared mixture was pipetted to the culture dish and rapidly transferred to the incubator for cross-linking. The cell-laden nanocomposite hydrogel was crosslinked after 1 h of incubation and cultured in the growth medium.

### 2.6 Cell viability assay

The effect of surface modification on cytocompatibility was evaluated with 2D cell culture by seeding cells with a density of 6000 cells/cm^2^ in 96-well plates. Cells were left to attach overnight, and the following day medium was replaced with nanoparticle-containing medium with the final concentration range of 10–100 μg/mL after 15 min sonication and vortexing. After incubation (24 h–72 h), Alamar Blue was added into wells as 10% of the sample volume, followed by 2 h of incubation at 37 ᵒC. The resulting fluorescent intensity was measured by using 550 nm excitation and 590 nm emission wavelength by using the Thermo Scientific Varioskan Flash plate reader. The average fluorescence values of the cell culture medium alone (background) were subtracted from the fluorescence values of experimental wells and vehicle (HEPES) treated cells were normalized to 100% viability for each time point.

The cell compatibility of MSN in 3D was assessed by preparing nanocomposite hydrogel that contained C2C12 cells with a concentration of 5x10^5^ cells/mL and MSN concentration ranging from 100 to 500 μg/mL. Stock solutions of MSN were prepared in HEPES buffer with a concentration of 5 mg/mL, dispersed by sonication, and introduced to the mixture before gelation. In 96-well plates, 50 μL of each sample was pipetted, incubated for crosslinking, and matrices were cultured in 150 μL media for 48 h. Resazurin reduction by metabolically active cells was evaluated by Alamar Blue assay as described previously (n = 3).

### 2.7 Intracellular uptake of MSNs in 2D cell culture and cell-laden nanocomposite matrix

For 2D cell culture, C2C12 cells were seeded to 6-well plates with a density of 4000 cells/cm^2^, incubated overnight for attachment, and the cell medium was replaced with 10 μg/mL MSN-containing medium the following day. T-MSN stocks were prepared as described previously. Cells were incubated to allow nanoparticle internalization. Cells were washed with PBS to remove non-internalized MSN, collected after trypsinization, and further washed twice. The fluorescence intensity corresponding to the endocytosed nanoparticles in cells was analyzed by BD LSR Fortessa flow cytometry instrument using excitation 561 nm and the Pe-Texas-Red channel was used for detecting signals. The data were analyzed with FlowJo v10 software (n = 3).

To evaluate the effect of surface modification on cellular internalization in 3D, cell-laden (2x10^6^ cell/mL) nanocomposite hydrogels were prepared containing 50 μg/mL T-MSN. After each time point, cultured substrates were collected in centrifuge tubes and dissociated by 2 mg/mL collagenase with 40 min incubation at 37 ᵒC. The collagenase working solution was prepared according to the protocol provided by the supplier. Gel and collagenase solution was pipetted after 20 min of incubation to facilitate faster dissociation of the collagen gel. Once the gel was fully dissolved, cells were collected by centrifugation and washed twice with cold PBS. Fluorescence detection resulting from nanoparticle internalization was performed by flow cytometry.

MSNs were dispersed in HEPES buffer at a concentration of 25 μg/mL and the fluorescence intensities were recorded within the identical excitation and emission spectral range employed by the flow cytometer. The mean values derived from two independent measurements were considered, and all intensity values were subsequently normalized by dividing them by the lowest recorded value, resulting in an arbitrary unit of 1. Then, these normalized values were subsequently employed for normalizing cellular uptake studies conducted using flow cytometry. Fluorescence intensity values of MSNs were included in the supplementary information (Table S1).

### 2.8 Nanoparticle distribution, cellular internalization, and DiO release by confocal microscopy

A total of 10 μL of neutralized and T-MSN incorporated (100 μg/mL) collagen solutions were pipetted to the μ-Slide angiogenesis dish (Ibidi) and incubated for 1 h at 37 ᵒC with 5% CO_2_. Cell medium was added on top of the crosslinked nanocomposite hydrogels, and nanoparticle distribution within the constructs was analyzed by using confocal microscopy with a displayed volume of 400 μm × 400 μm x 250 μm using Zeiss LSM880 with Airyscan (excitation: 543 nm HeNe laser, emission: 560–580 nm), 20x objective. Representative Z-stack images captured by confocal microscopy were reconstructed into a 3D image using the Zeiss Zen Black 2 software.

Microscopy images for 2D and 3D cell cultures were prepared similarly to flow cytometry experiments. For 2D cell culture microscopy samples, sterile coverslips were placed in 6-well plates, and cells were cultured on coverslips. After nanoparticle treatment for 24 h, cells were stained with CellMask™ deep red plasma membrane stain for 10 min followed by washing with PBS and fixation with 4% PFA at room temperature. Coverslips were washed three times with PBS and mounted with VECTASHIELD with DAPI.

For nanoparticle internalization detection in cell-laden nanocomposite hydrogels, cells were fixed with 4% PFA at room temperature for 30 min, washed with PBS three times, and permeabilized with 0.1% Triton X-100 in PBS for 60 min at RT. F-actin was stained for 1 h at RT with Alexa Fluor Phalloidin 633 nm solution (165 nM) containing 1% BSA. 3D matrices were further stained with nucleus staining DAPI (1 μg/mL) in PBS for 10 min at RT, followed by washing step with PBS three times between each step. Samples were soaked in PBS and stored at 4 °C until confocal microscopy acquisition. Images were acquired by Zeiss LSM880 with Airyscan. (DAPI ex: 405 nm, em:410–540 nm, TRITC ex:543 nm, em:550–620 nm, CellMask/Phalloidin Ex:633 nm, Em: 640–750 nm)

The surface modification effect of nanoparticles on drug delivery in the 3D matrix was evaluated by pipetting T-MSN/DiO nanoparticles (50 μg/mL) into 150 μL of collagen solution and then crosslinking by incubation in 35 mm imaging dishes (Ibidi). Additionally, the gels were washed with 2 mL of cell medium, and the washing medium was then used to treat cells grown on coverslips to assess the stability of MSN-collagen interaction. Cells were seeded on top of the nanocomposite hydrogel with a density of 4000 cells/cm2 and incubated at 37 ᵒC and 5% CO_2_ and live-cell microscopy images were acquired at 4, 24, and 48 h time points. Nanoparticle internalization and the release of the hydrophobic drug model were imaged by confocal microscopy. Meanwhile, the cells growing on glass coverslips in the washing medium were fixed after 24 h incubation and the internalization of MSN which was released from the nanocomposite hydrogel was analyzed by confocal microscopy.

### 2.9 Live-cell imaging

The cells were seeded onto 12-well plates at a density of 5000 cells/cm^2^ and cultured overnight for attachment. On the following day, the cell medium was replaced with the medium containing 25 μg/mL T-MSN/DiO, and cells were placed in the IncuCyte S3 live-cell imaging instrument. Images were acquired with phase contrast (cells), green channel (DiO), and red channel (T-MSN), and samples were imaged for 48 h at 2 h intervals and with a total of nine images per sample. The acquired data was plotted as a function of time, determining whether DiO release from T-MSN corresponded with an increase in the green area (n = 2).

### 2.10 Notch inhibition studies in 2D and 3D cell cultures

Myoblasts were treated with empty nanoparticles with a concentration of 100 μg/mL for 48 h in 2D culture and the gene expression levels of Hes1 and Hey1 were assessed to study the possible interference of MSN alone on Notch signaling.

The response to Notch inhibition in ligand-induced cells was tested on C2C12 myoblasts cultured in a 12-well plate coated with recombinant Dll4-Fc chimera (R&D Systems) or Fc-IgG (Jackson ImmunoResearch) at concentrations of 10 nM. Plates were coated with 50 μg/mL Protein G (Zymed), blocked with 1% BSA, and further coated with ligands as described previously [57]. Cells were seeded with 4500 cells/cm^2^ density and harvested for qPCR analysis after treatment with 10 μM DAPT-containing medium for 48 h. Acquired data represented 3 independent experiments.

Before the evaluation of the MSN-mediated Notch inhibition and myogenic commitment in 2D cell culture, DAPT amount in each MSN sample was determined by HPLC measurement. A stock solution of DAPT-loaded MSN was prepared in HEPES buffer containing 100 μM DAPT by using ultrasonication (MSN concentration varied depending on the loading degree and surface modification). Cells were treated with MSN-DAPT, or free drug (5 μM DAPT concentration), and their corresponding vehicle solvents were used as control. DAPT was dissolved in DMSO as a free drug and the final concentration of DMSO in cell studies was kept below 0.1% to avoid cytotoxicity. After incubation, cells were lysed to proceed with RNA isolation and qPCR.

To study the MSN-mediated Notch inhibition in nanocomposite hydrogel, MSN-DAPT stock solutions were prepared similarly. 5 μM DAPT-containing free DAPT or MSN-DAPT was incorporated within the 200 μL collagen along with C2C12 myoblasts (2 x10^6^ cells/mL) to create cell-laden nanocomposite hydrogel in 48-well plates. 3D matrices were transferred to 24-well plates using sterile flat-tip forceps after 2 days of incubation to allow larger volume capacity for cell media. Medium was replaced every second day and samples were collected after day 2 (48 h) and day 7 (168 h) incubation. mRNA expression levels of Notch target genes were analyzed by qPCR. For both 2D and 3D *in vitro* cell cultures, MSN-DAPT was introduced to cells as a bolus, whereas free drug samples were re-treated with DAPT every second day.

### 2.11 RNA isolation and quantification of RNA expression by quantitative real-time polymerase chain reaction (qPCR)

NucleoSpin RNA kit (Macherey-Nagel) was used to isolate total RNA from cells for 2D and 3D cell cultures to measure the Notch inhibitory effect of DAPT by MSN-facilitated drug delivery and differentiation markers. The purity of the RNA was determined using NanoDrop spectroscopy (Thermo Scientific). Samples displaying 260/280 ratios greater than 2.0 and 260/230 ratios greater than 1.8 were considered acceptable and lower ratios were repurified by using the same kit. For cDNA synthesis, 0.5 μg of total RNA was used using the SensiFast kit (Bioline) as described by the manufacturer. cDNA preparations were then diluted 20-fold before PCR analysis, and the same preparations were used throughout the studies. Real-time PCR was performed using HOT FIREPol EvaGreen qPCR Mix Plus following a protocol provided by the supplier. The thermal amplification was conducted by QuantStudio 3 instrument (Thermo Fisher). The calculated quantity values with the performance of the primer efficiency were exported. The RNA expression levels were normalized against the housekeeping gene Rpl13a and compared with the control sample, which was set arbitrarily at 1.0 to indicate fold change. Primers to Hes1, Hey1, Mef2a, Myogenin, Myf5, and Myh4 were designed using primerBLAST to produce mouse-specific amplicons of 80-200 bp, spanning one or more exon/exon boundaries to prevent amplification of trace genomic DNA carryover. The sequences of the forward and reverse primers are listed in the supplementary section. All experiments were performed with three biological replicates, i.e., three independent experimental procedures. A melting curve analysis was performed to verify that the product consisted of a single amplicon. Forward and reverse primer sequences can be found in Table S2.

### 2.12 Statistical analysis

Data are presented as the mean of three independent experiments with standard error of the mean (SEM). Statistical analysis was performed by GraphPad Prism (9.0.0) software. Two-tailed, unpaired student's t-test was used to evaluate the significance between two groups, and one-way ANOVA with Tukey post-hoc was used to investigate the significance when more than two groups were present. A difference with a p-value less than 0.05 was considered statistically significant.

## 3 Results and discussion

### 3.1 Synthesis and characterization of differently functionalized MSNs

To formulate a biocompatible and bioactive MSN-based nanocomposite hydrogel, MSNs were synthesized via the co-condensation method with the aminosilane APTES as a co-silica source to achieve amine groups on the pristine nanoparticles that support further modifications. The obtained MSNs were then further modified with polyethylene imine (MSN-PEI) to enhance electrostatic stabilization and enable further surface charge tuning [48]. Thus, to assess the role of surface charge in nanocomposite hydrogels, the hyperbranched PEI layer was alternatively capped with acetic anhydride to provide a near-net neutral surface charge (MSN-PEI-ACA); derivatized with succinic anhydride, yielding terminal carboxylic acid groups on the PEI layer (MSN-PEI-SUC); or modified with polyethylene glycol (methyl ether) (mPEG) polymer to increase the steric colloidal stabilization by the non-ionic hydrophilic polymer [58]. Overall, secondary modifications are further implemented to enhance the complexity on the surface groups of MSN-PEI while preserving the PEI modification, since PEI also carries secondary and tertiary amines and SUC/ACA/PEG modification only binds to primary amines.

**Fig. 1:**
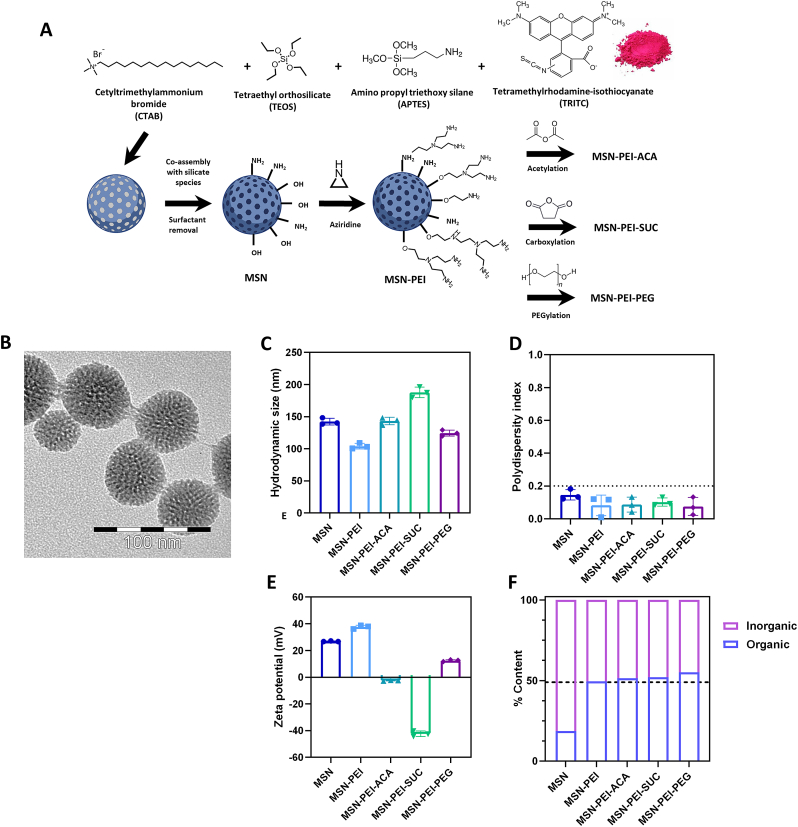
Physicochemical characteristics of MSNs. **(A)** Schematic illustration of nanoparticle synthesis and surface modifications of MSNs. **(B)** Transmission electron microscopy (TEM) images reveal the porous structure of MSNs. **(C**–**D)** Dynamic light scattering (DLS), and **(E)** ζ-potential measurements of surface-modified MSNs as measured in 25 mM HEPES buffer (pH 7.2) with a concentration of 0.1 mg/mL. **(F)** Thermogravimetric analysis (TGA) shows surface modification percentage as organic content in proportion with silica as inorganic content.

Nanoparticles are often studied by tracing them with fluorescent dyes and thus, stable dye incorporation is necessary to avoid the possibility of dye leakage, which can lead to incorrect interpretations [59]. Therefore, fluorescent dye TRITC was incorporated directly into the synthesis to achieve inherently fluorescent nanoparticles for fluorescence-based biological experiments such as microscopy and flow cytometry. Since TRITC is an amine-reactive dye, it can covalently bind directly to the amine group of APTES in the synthesis process. Fig. 1A demonstrates the schematic illustration of the surface modification process of MSNs.

Surface-modified MSNs were characterized by transmission electron microscopy (TEM), dynamic light scattering (DLS), ζ-potential measurements, and thermogravimetric analysis (TGA). The morphology and mesoporous structure of the MSN was visualized by TEM and the observed size was approximately 55 nm with radial pore distribution (Fig. 1B, Fig. S1). Dynamic light scattering (DLS) measurements revealed all surface-modified MSNs to be unimodal size distribution with an average hydrodynamic diameter of 140 nm (Fig. 1C). Here we note that a discrepancy between DLS and TEM is to be expected, due to the fact that our particles are both fluorescent and porous, both known to deviate the DLS readout. While fluorescent samples may render the DLS results unreliable, porous particles increase the deviation between DLS and TEM to a significant degree [[60], [61], [62]]. Therefore, for porous solid particles such as MSNs, TEM is to be regarded as a more reliable size measure while DLS is mostly employed to ensure full (re)dispersibility in the solvent phase. Not surprisingly regarding (re)dispersibility, PEI modification on the MSN surface slightly reduced the polydispersity index (PDI) values while subsequent ACA, SUC, and PEG modifications did not further impact the results, suggesting that MSNs maintain full redispersibility and hydrodynamic size distribution following successful surface functionalization (Fig. 1D). Co-condensation with APTES in the synthesis of MSNs resulted in an average ζ-potential of +26 mV in HEPES buffer (25 mM, pH 7.2). PEI modification increased the net surface charge to +38 mV under the same conditions. ACA modification capped the primary amine groups of PEI resulting in a near neutral charge of −2 mV, whereas carboxylation of the amine groups of PEI (MSN-PEI-SUC) resulted in a strong net negative charge, −42 mV at neutral pH. PEG modification on top of MSN-PEI resulted in a decrease in net positive charge and thus, the ζ-potential decreased to +13 mV (Fig. 1E). TGA measurements were performed to validate the successful surface engineering of MSNs. The results revealed 18.7% weight loss for MSN due to amino group functionalization and 49.6% weight loss for MSN-PEI, indicating the high surface grafting density. Further surface modifications were validated with only slightly increased weight loss to 51.4% for ACA, 52.1% for SUC, and 55.1% for PEG (Fig. 1F).

### 3.2 Evaluation of surface-modified MSNs in cytocompatibility and cellular internalization in 2D culture

**Fig. 2:**
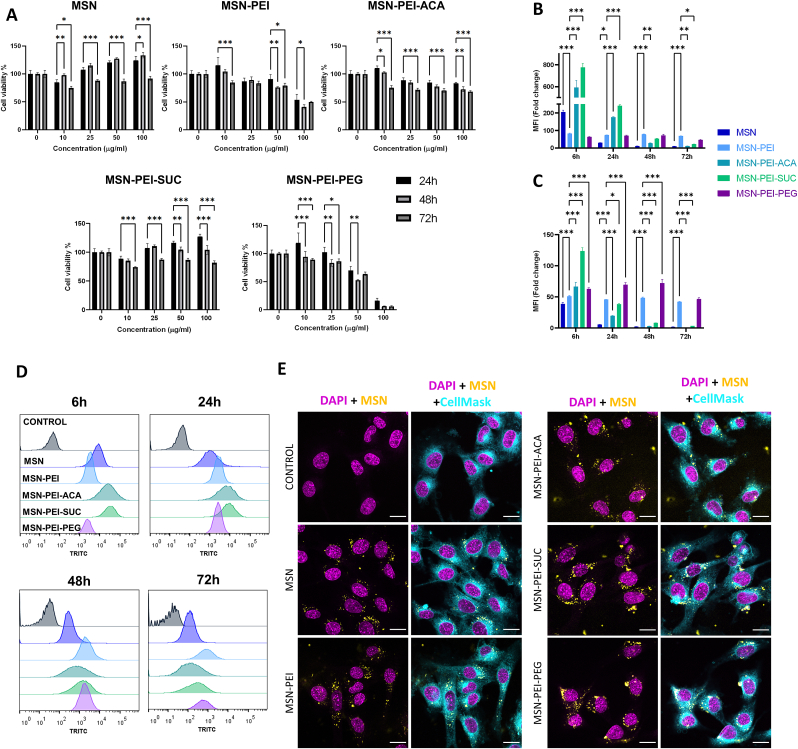
Cytocompatibility and internalization of surface-modified MSN with C2C12 myoblasts in 2D culture. **(A)** Cell viability was measured with Alamar Blue after incubation with surface-modified MSNs (10, 25, 50, 100 μg/mL) for 24, 48, and 72 h (n = 3, mean ± SEM). **(B)** Mean fluorescence intensity (MFI) of internalized MSNs (10 μg/mL) acquired by flow cytometry after incubation for 6, 24, 48, and 72 h (n = 3, mean ± SEM). **(C)** Normalized MFI of flow cytometry results according to MSN intensity. **(D)** Histogram charts of cellular uptake indicate the frequency distribution of different surface modifications for different time points. **(E)** Confocal microscopy images of MSNs (10 μg/mL) after 24 h incubation. Differences between samples were analyzed using two-way ANOVA with Dunnet's post-hoc, *p < 0.05, **p < 0.01, ***p < 0.001. (For interpretation of the references to color in this figure legend, the reader is referred to the Web version of this article.)

Mouse C2C12 skeletal muscle precursor cells (also called myoblasts) are commonly used for biomaterial research for skeletal tissue engineering, due to their capability of differentiating rapidly, forming contractile multinucleate myotubes, and exhibiting characteristic muscle proteins [63,64]. To develop a novel nanocomposite hydrogel with the desired functionality, we assessed the biocompatibility and internalization of the differently functionalized MSNs. To study the role of surface engineering in biological behavior, C2C12 cells were cultured with surface-modified MSNs and cell viability was investigated by Alamar Blue assay as a function of dose and time of the treatment (Fig. 2A). The mesoporous silica being non-toxic, low concentrations of MSN demonstrated cell viability enhancing properties. In all cases except MSN, cytotoxicity was only observed at over 50 μg/mL concentrations with longer incubation time, suggesting high tolerance to MSNs, considering the initial low seeding density. Surprisingly, MSN-PEI-PEG was found to exhibit the highest cytotoxicity, although PEG has been known to be a non-toxic polymer [65]. MSN-PEI has shown the second-highest toxicity possibly due to having cationic charge density, which leads to strong interaction of PEI with the cell membrane that causes disturbance [66]. However, secondary ACA and SUC modifications improved the cytocompatibility of PEI modification and were tolerated by cells with only a 20–30% decrease in cell viability at 100 μg/mL even after 72 h, suggesting that ACA and SUC shielded the cationic amine groups of PEI.

The effect of surface modification on cellular internalization was evaluated by using TRITC-labelled MSNs with flow cytometry (Fig. 2B–C) after incubation over a time period (6, 24, 48, 72 h) with a biocompatible dose of MSNs (10 μg/mL). Mean fluorescence intensity (MFI) results indicated that PEI modification decreased the internalization; however, when the fluorescence intensities were normalized according to nanoparticle fluorescence intensity, PEI modification enhanced the cellular uptake, as reported previously [46]. ACA, SUC, and PEG surface modifications on MSN-PEI led to further enhancement in cellular internalization for 6 h. Overall, quantitative analysis of MFI in the MSNs treated cells showed high intensity with the maximum signal at 6 h, indicating high cellular internalization of C2C12 cells, followed by a decremental pattern over time. These results imply nanoparticles were efficiently shared between daughter cells as they divide, allowing longer duration for intracellular drug delivery, as previously reported [[67], [68], [69]].

Histogram charts of cellular uptake in Fig. 2D indicate the overall distribution spread over time which supports the hypothesis. Additionally, ACA modification shows a larger spread even at a 6 h time point, which indicates that nanoparticles were internalized with a higher variation that could result from the lack of charge repulsion and possible aggregation. According to confocal microscopy images of cells treated with MSNs for 24 h (Fig. 2E), MSN and PEI nanoparticles were found to maintain their colloidal stability and internalized and appeared as smaller clusters in cells, whereas ACA, SUC and PEG nanoparticles were found to form larger aggregates.

### 3.3 Evaluation of cytocompatibility and cellular internalization in 3D composite hydrogels

**Fig. 3:**
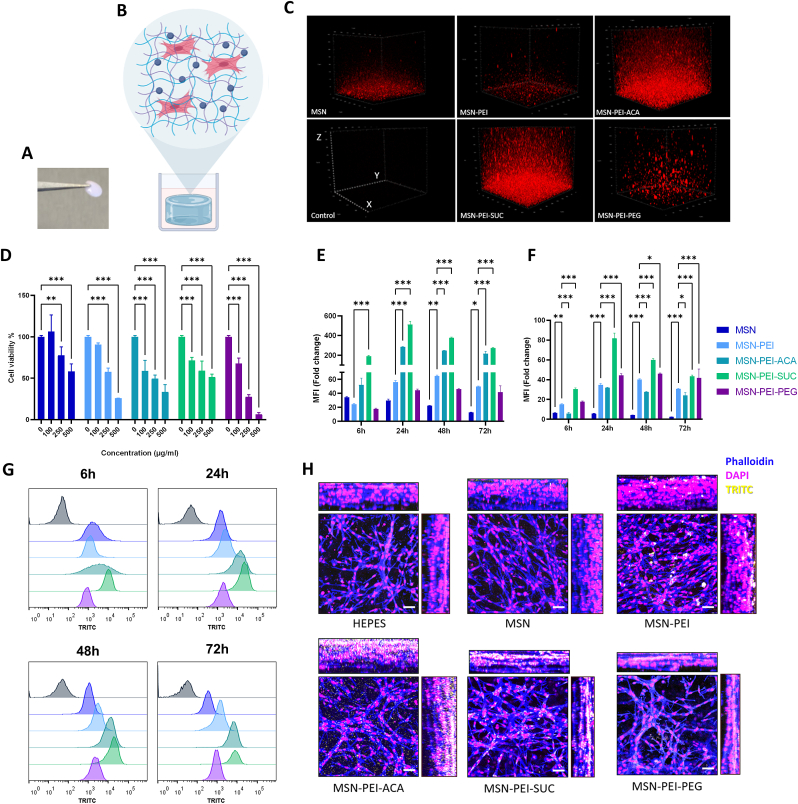
Evaluation of MSN surface modification influence on cytocompatibility and cellular internalization in the nanocomposite hydrogel. **(A)** Image of the cell-laden nanocomposite hydrogel after 48 h of incubation. **(B)** Schematic illustration of the nanocomposite hydrogel prepared by using collagen, surface functionalized MSN, and myoblast cells. **(C)** MSN distribution in the matrix was investigated using reconstructed volume (3D) images acquired by confocal microscopy with the voxel volume of 400 μm × 400 μm x 250 μm. **(D)** Cytocompatibility study of surface modified MSNs for C2C12 myoblasts in cell-laden nanocomposite hydrogel. Data represent the mean of 3 independent experiments ±SEM. The differences with respect to control were analyzed using one-way ANOVA, *p < 0.05, **p < 0.01, ***p < 0.001. **(E)** MSN uptake based on mean fluorescence intensity (MFI) values in nanocomposite hydrogel (3D matrix) obtained from flow cytometry and **(F)** normalized MFI values according to MSN fluorescence intensity. Differences between samples were analyzed using two-way ANOVA with Dunnet's post-hoc, *p < 0.05, **p < 0.01, ***p < 0.001. **(G)** Histogram charts of MSN uptake indicate the frequency distribution of different surface modifications for different time points in 3D. **(H)** Orthogonal view of cell-laden nanocomposite hydrogel containing 50 μg/mL MSNs after 24 h incubation showing the spatial distribution of nanoparticle internalization within the cells. Images represent F-actin staining with phalloidin (blue), nuclei staining with DAPI (magenta), and TRITC-MSNs (yellow), internalized MSN (=colocalization of F-actin and TRITC-MSN, white). The scale bar corresponds to 50 μm. (For interpretation of the references to color in this figure legend, the reader is referred to the Web version of this article.)

In tissue engineering, intracellular drug delivery can be used to improve the microenvironment provided by material scaffolds and to increase the molecular signaling of cells within these microenvironments [70]. Collagen fibrils allow biomolecules to be covalently or non-covalently attached into a collagen scaffold via molecular interactions owing to charged and nonpolar amino acid residues; hence, it is suitable for nanocomposite hydrogel development [71]. Collagen was chosen as the preferred matrix to explore nanocomposites as a consequence of its ease of preparation, being a natural polymer, and possessing translational relevance in the biomaterials field. After confirming the biocompatibility of MSNs in 2D culture and achieving intracellular internalization, surface modified MSNs were incorporated in neutralized collagen solution to formulate the nanocomposite hydrogel. Cells were further incorporated in the blend, and cell-laden nanocomposite hydrogels were achieved by incubating at 37 °C. Forceps were easily capable of handling the cell-laden nanocomposite hydrogels after culturing for 48 h (Fig. 3A–B). Volume (3D) images of MSN-hydrogel composites were acquired with confocal microscopy to assess MSN distribution profiles and homogeneity within the matrix with the aid of TRITC fluorescence (Fig. 3C). Based on the results, MSN-PEI-ACA and MSN-PEI-SUC showed the best distribution profile whereas MSN, MSN-PEI, and MSN-PEI-PEG showed aggregation with uneven distribution. Cluster formation on MSN-PEI-PEG containing hydrogel was not unexpected as Fig. 2E indicated self-aggregation of nanoparticles when the serum-containing medium was introduced. On the contrary, strong interactions of nanoparticle-collagen for MSN-PEI might have led to interparticle aggregation within the composite hydrogel [72]. ACA and SUC modifications seemed to have less interaction with collagen fibers allowing them to maintain their colloidal form within the matrix, resulting in a homogenous nanocomposite also during cross-linking. Nanoparticles with available amino groups exhibiting poor homogeneity could be attributed to the strong interaction with negatively charged groups on collagen fibers prior to the crosslinking process [73]. Additionally, our previous studies showed SUC modification performs superior in terms of circumventing protein corona compared with PEI and PEG modifications, suggesting stronger interactions might have occurred between MSN and PEI and collagen fibers, resulting in rapid aggregation [48]. On the other hand interparticle agglomeration of PEG modified MSNs might be responsible for the non-homogenous distribution in nanocomposite matrix.

Cytocompatibility studies in 3D cell culture demonstrated dose dependent toxicity (100–500 μg/mL) for all surface modified MSNs with a higher tolerance for 100 μg/mL concentration, where more than ≈59% of the cells remained viable after 48 h of incubation (Fig. 3D). Overall, when cells were treated with 100 μg/mL, plain MSN showed no cytotoxic effect whereas MSN-PEI containing matrices exhibited ≈91% cell viability. Secondary modifications MSN-PEI-ACA, MSN-PEI-SUC, and MSN-PEI-PEG slightly reduced the cytocompatibility with cell viabilities of ≈59%, ≈72%, and ≈68%, respectively. In connection with nanoparticle distribution data, surface modifications that resulted in high homogeneity within the hydrogel were also found to have a higher cellular internalization due to their increased accessibility throughout the matrix (Fig. 3E and G). Our results showed that nanoparticle internalization in a 3D matrix occurred at a slower rate than in 2D culture studies, where the highest rate of internalization was observed at the 24 h time point. According to normalized MFI (Fig. 3F), MSN-PEI-SUC showed the highest cellular internalization. This finding can be explained by the nanoparticles being negatively charged, whereby the electrostatic interactions between MSN-PEI-SUC and the charged components of collagen fibers may have contributed to the repelling effect, retaining the nanoparticles readily available for cells. Histogram charts for cellular internalization in 3D culture showed similar results as Fig. 2D, with MSN-PEI-ACA having the largest spread at the earlier time point (Fig. 3G). Based on the visual inspection of the orthogonal view of 3D composite hydrogels, the highest level of internalization was observed in the hydrogel containing MSN-PEI-SUC nanoparticles (Fig. 3H, Fig. S2). On the other hand, MSN-PEI-ACA composite hydrogel contained a significant amount of non-internalized nanoparticles after 24 h, indicating slower nanoparticle internalization. Large aggregates of MSN-PEI were observed in hydrogels in Fig. 3H, suggesting that positively charged MSN-PEI modification is not suitable for nanoparticle-collagen composite hydrogel formulation, and net negative charged SUC, or net neutral charged ACA nanoparticles are more suitable for nanoparticle mediated drug delivery in 3D composite hydrogels.

### 3.4 Evaluation of hydrophobic drug delivery of MSNs in 2D and 3D cultures

**Fig. 4:**
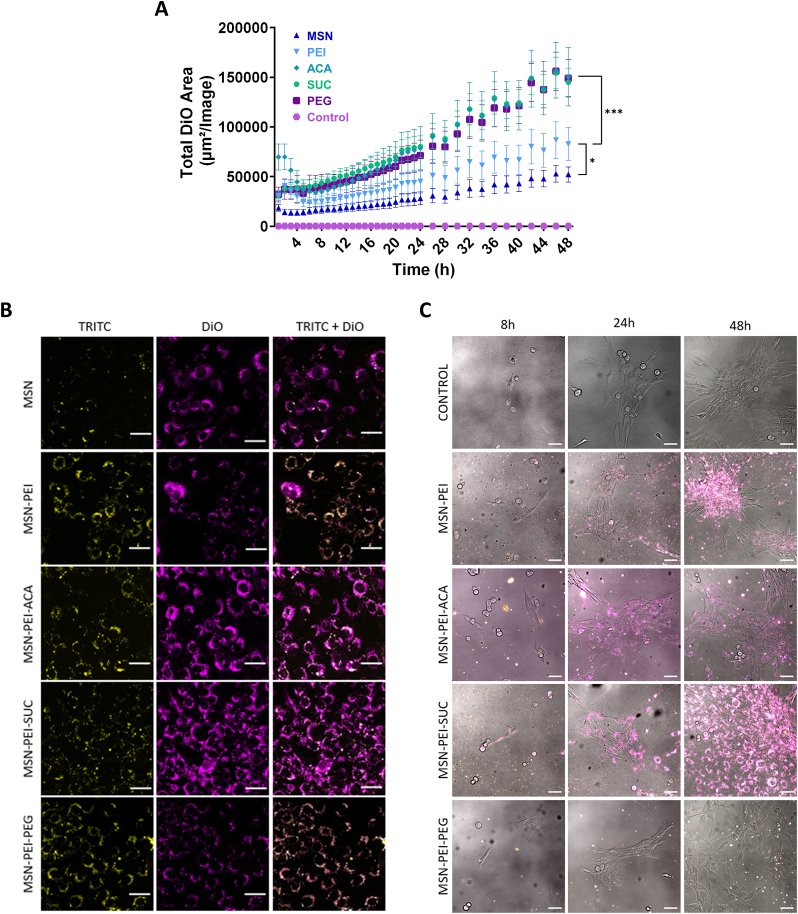
Evaluation of the role of the surface modification in drug delivery using a hydrophobic model drug (DiO) in 2D and 3D cell cultures set-up. **(A)** Total DiO fluorescence area over time depicts the intracellular release acquired by the IncuCyte live-cell analysis system. Data are shown as mean ± SEM of duplicate wells and are representative of two independent experiments analyzed with one-way ANOVA with Bonferroni post-hoc: *p < 0.05, ***p < 0.001. **(B)** Confocal microscopy images of myoblasts after 48 h incubation with 25 μg/mL containing T-MSNs/DiO in 2D culture demonstrate the internalized MSNs and released DiO. The scale bar is 40 μm. **(C)** Time series of confocal microscopy images of myoblasts cultured on T-MSNs/DiO incorporated hydrogels (2.5D) exhibiting nanoparticle internalization from hydrogels and intracellular release. Composite images consist of overlayed phase contrast (grey), TRITC (yellow), and DiO (magenta). The scale bar corresponds to 40 μm. (For interpretation of the references to color in this figure legend, the reader is referred to the Web version of this article.)

MSN as a drug delivery vehicle is favored due to the possibility to achieve improved stability of incorporated agents, enhanced solubility of hydrophobic drugs, confined release of the drug at the target site, and thereupon increased drug efficacy [19,54]. The rigid inorganic framework of MSN offers resistance to environmental influence, in addition to the large surface area and the large number of pores in MSN allowing for high drug loading and a higher rate of drug release, which makes it particularly attractive for delivery of poorly water-soluble drugs [74]. We therefore explored the role of surface modification of MSN on drug delivery to myoblasts using inherently TRITC-labelled nanoparticles loaded with the hydrophobic model drug DiO to allow detection of both localization of MSN and hydrophobic drug delivery by fluorescence (Fig. 4). Low loading degree was aimed for according to the previous studies that allowed sustained release [68,75] and the final calculated loading degree of nanoparticles was similar regardless of surface modification, ranging between 2 and 3 wt% (Figs. S3A–S3B), as observed previously [76]. Live-cell imaging was performed to avoid the possible interference of dye release due to cell fixation. As shown in Fig. 4A, the analysis of the DiO area over time revealed that secondary modifications of ACA, SUC, and PEG led to a faster intracellular release of DiO when compared to MSN and MSN-PEI. A similar release profile was observed for SUC and ACA, even though ACA modification led to a higher initial DiO area indicating that aggregation is taking place in the cell medium, possibly due to its near-neutral net surface charge. IncuCyte imaging at 48 h of incubation was consistent with the data in Fig. 4A, demonstrating a larger area of green fluorescence (indicating endosomal escape and intracellular release) for ACA, SUC, and PEG modifications (Fig. S4).

The TRITC fluorescence appears to have bleached over time probably due to repeated exposure, and therefore nanoparticle internalization and intracellular release could not be detected simultaneously by the IncuCyte live-cell analysis system. Confocal microscopy was utilized instead for detailed analysis of internalization and release from the nanoparticles after 48 h incubation. As depicted in Fig. 4B, the highest intracellular release (magenta, DiO channel) was achieved by SUC and ACA-modified MSNs. Even though PEG modification showed widespread distribution in the DiO channel, direct colocalization with the TRITC channel (yellow) implies intracellular release requiring longer periods. Similarly, PEI modified nanoparticles had strong colocalization with the TRITC channel and a smaller spread of intracellular release. Our previous reports have pinpointed the presence of MSN-PEIs in endosomes at 72 h after incubation supporting the necessity of the longer timeframe for intracellular release [77] and the release rate can be further promoted by utilizing ACA or SUC modification for more efficient cargo delivery in a shorter period in this case.

To investigate the intracellular delivery efficiency of MSN in a more biologically relevant setting, cells were cultured in a 2.5D model, consisting of a thick layer of nanoparticle-hydrogel composite that recapitulates some characteristics of the microenvironment still allowing the benefit of single plane imaging, which minimizes the duration of imaging and reduces phototoxicity [78,79]. Intracellular release of DiO-loaded MSN-PEI-SUC and MSN-PEI-ACA nanoparticles was observed already at 24 h (Fig. 4C). The release was sustained over the period of 48 h. MSN-PEI nanoparticles demonstrated a slower release profile, whereas MSN-PEI-PEG did not show any intracellular release up to 48 h, likely due to poor particle internalization (Fig. 3E–H). To study if nanoparticles escape from the hydrogels, composite hydrogels were washed with cell media, the media was collected and used to treat cells for 48 h. According to confocal microscopy images, no nanoparticle internalization or intracellular release was observed, indicating that the hydrogel acts as a reservoir for the nanoparticles and prevents the nanoparticles from escaping (Fig. S5). Taken together, our results suggest a similar trend can be observed between 2D and 2.5D in terms of intracellular release and it is highly dependent on the cellular internalization rate. MSN-PEI-ACA and MSN-PEI-SUC nanoparticles proved to be the most effective in delivering their cargo to myoblasts when combined with hydrogel, thus making them solid candidates for drug delivery applications in 3D culture systems.

### 3.5 MSN-mediated Notch inhibition in 2D culture

**Fig. 5:**
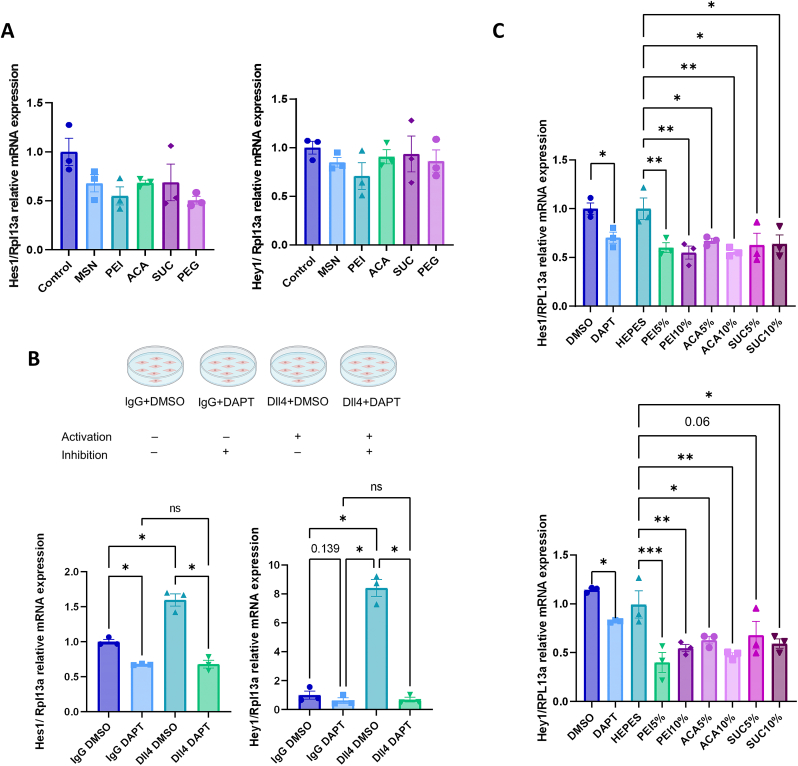
Transcriptional response to Notch signaling inhibition in C2C12 cells. **(A)** Cells were exposed to surface-modified empty nanoparticles for 48 h, and the expression of Notch signaling target genes was quantitatively determined by qPCR. **(B)** Gene expression in response to ligand induction and DAPT treatment measured by qPCR after 48 h. **(C)** Gene expression in response to the DAPT-loaded nanoparticles with different loading degrees treated with the final concentration of 5 μM DAPT. Differences between samples were analyzed using one-way ANOVA with Tukey's post-hoc, *p < 0.05, **p < 0.01, ***p < 0.001.

Given the known role of Notch signaling in regulating the fate of myoblast cells [80,81], we hypothesized that we could inhibit the Notch signaling pathway by employing MSNs to deliver DAPT to myoblast cells in a temporally and spatially controlled manner. Before studying the effect of inhibition with DAPT, C2C12 myoblasts were treated with empty MSNs, and their influence over Notch target gene expressions (Hes1, Hey1) was determined to establish the impact of the nanocarriers themselves on Notch activity (Fig. 5A). There was no significant difference in the expression levels of Hes1 and Hey1 upon treatment with MSNs, although a slight decrease was observed in Hes1 expression levels. We have also previously studied the possible side effects of MSNs on differentiation and no adverse effects were observed on the expression of myogenic proteins [82].

Next, we evaluated the efficiency of DAPT in inhibiting Notch signaling for C2C12 myoblasts on the basal level (cells treated with recombinant Fc-IgG ligand) and induced Notch activity level (cells treated with recombinant Fc-Dll4 ligands) by exposing cells to DAPT for 48 h qPCR results revealed that DAPT was able to significantly inhibit Notch signaling as demonstrated by the downregulation of Hes1 and Hey1 expression. Although the level of downregulation in the cells with basal Notch activity seemed relatively small (<2-fold for Hes1 and approx. 2-fold for Hey1), there was no significant difference in the expression levels between the cells with basal and induced Notch activity upon DAPT treatment. This suggested that basal levels of Notch signaling in these cells are sufficient to stringently study Notch inhibition (Fig. 5B). Furthermore, to find the optimal dose of DAPT, expression levels of Hes1 and Hey1 were evaluated with respect to DAPT concentration. Based on the results, a DAPT concentration of ≥5 μM was sufficient to inhibit Notch signaling in C2C12 myoblasts (Fig. S6). These findings are consistent with those previously reported [83].

To investigate the impact of the loading degree on Notch inhibition, PEI, ACA, and SUC surface-modified nanoparticles were loaded with two different loading degrees of DAPT (5% or 10%), Cells were treated with drug-loaded nanoparticles with the final DAPT concentration equivalent to 5 μM in cell medium, and this translated to the following concentrations of MSNs applied to cells as final concentration in cell media: 0.11 mg/mL for PEI 5%, 0.09 mg/mL for ACA 5%, 0.15 mg/mL for SUC 5%, 0.07 mg/mL for PEI 10%, 0.05 mg/mL for ACA 10%, and 0.04 mg/mL for SUC 10%, respectively. Further experiments to verify the functionality of DAPT-loaded MSNs confirmed that the expression of Hes1 and Hey1 was downregulated by MSNs to a similar degree as free DAPT, regardless of the loading degree and surface modification (Fig. 5C). Therefore, to carry out further 3D culture experiments, the results relating to cellular viability, cellular internalization, and the delivery efficiency of DiO were considered, and MSN-PEI-SUC nanoparticles with a 10% loading degree were selected. We have then evaluated the Notch inhibition effect of DAPT-loaded MSN-PEI-SUC in 2D culture at three different time points (0, 24 h, and 48 h) (Fig. S7) and achieved a Notch inhibitory effect with nanoparticles after 48 h, suggesting a delayed drug response mediated by nanoparticles. The delayed inhibitory effect may provide a beneficial acclimation period for cells to adapt to the nanocomposite hydrogel environment in a tissue repair context before being exposed to cell-fate-inducing signals.

### 3.6 Evaluation of designed nanocomposite hydrogel on Notch inhibition and myogenic differentiation

DAPT inhibition response in 3D matrices was evaluated by exposing the cell-laden hydrogels with a single dose or repeated doses of DAPT. Notch target genes and markers of myogenic differentiation were measured at an early (two days) and a late (seven days) time point by qPCR (Fig. S8). Consistent with the results of the 2D cell culture studies, Hes1 and Hey1 expression was downregulated by approximately two folds after 2 days of DAPT treatment compared to DMSO, whereas the expression of the marker genes for myogenic differentiation (Mef2a and Myogenin) remained unchanged. The initial single dose of DAPT treatment did not mediate a significant change in transcriptional response after 7 days. On the contrary, continuous DAPT treatment led to the downregulation of Hes1 and Hey1, and, albeit with no statistical significance, upregulation of the differentiation markers, in line with previous studies conducted on C2C12 myospheres [84] and human myogenic cells [85]. The findings suggest that transient inhibition of Notch signaling is not sufficient for promoting myogenic differentiation, and a continuous inhibition of Notch over extended periods is needed.

**Fig. 6:**
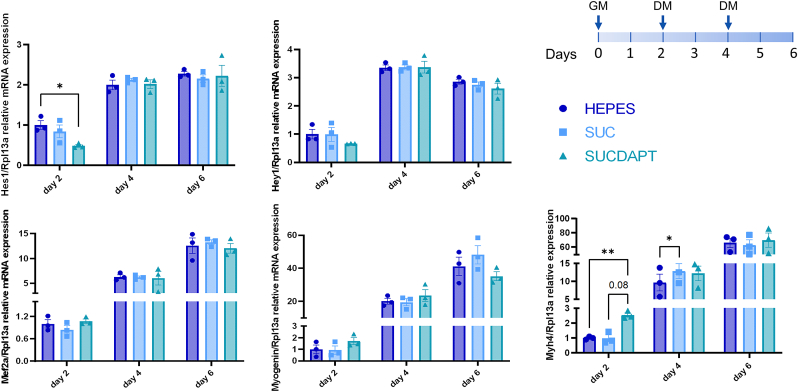
DAPT-containing cell-laden nanocomposite hydrogel efficiently inhibits Notch signaling and promotes myogenesis. Evaluation of mRNA expression levels of Notch signaling (Hes1, Hey1) and differentiation markers (Mef2a, Myogenin, Myh4) by the cells grown in nanocomposite hydrogels containing empty MSN-PEI-SUC or DAPT loaded MSN-PEI-SUC nanoparticles. HEPES was used as a vehicle to disperse nanoparticles. Each experiment was carried out with three independent experiments (mean ± SEM, n = 3). Two-tailed, unpaired student's t-test was used to evaluate the significance between two groups, and one-way ANOVA with Tukey's post-hoc was performed for comparison of more than two groups, *p < 0.05, **p < 0.01, ***p < 0.001.

Taking advantage of the delayed Notch inhibition with MSN-PEI-SUC, and the high nanoparticle internalization in the 3D matrix, we next assessed if the nanocomposite hydrogels would allow direct and controlled delivery of DAPT and drive myogenesis in the cell-laden 3D matrix. Samples were initially cultured in growth media for two days to allow cell proliferation and internalization of nanoparticles, after which differentiation was induced by differentiation media (Fig. 6). Samples were collected on day 2, day 4, and day 6, and the expression of Notch targets and myogenic differentiation markers were analyzed with qPCR. After 2 days, samples with DAPT-loaded MSN-PEI-SUC demonstrated downregulation of the Notch targets with similar efficacy to that achieved previously with free DAPT (approx. two folds), suggesting that the particles successfully delivered the drug. Surprisingly, there was already upregulation of the late myogenic marker myosin heavy chain 4 (Myh4) of DAPT-treated samples on day 2, whereas no significant difference was observed on early markers mef2a and myogenin; indicating nanocomposite hydrogels can rapidly alter cellular response in 3D matrices and nanoparticle-aided DAPT delivery may support differentiation. However, on days 4 and 6, all groups showed increased expression of differentiation markers, and no significant difference was found either in the expression of the differentiation markers or the Notch target genes between the groups, suggesting that the matrix-incorporated nanoparticles delivered DAPT transiently over the first two days. All confocal microscopy images showed a dense network after 6 days of incubation (Fig. S9). Overall, it might be challenging to capture the DAPT-mediated differentiation in this setting due to the differentiation-promoting effect of collagen and the biphasic nature of Notch activity in differentiating myoblasts [86]. Regardless, we demonstrated successful DAPT delivery in a sequential and temporal manner in the cell-laden nanocomposite hydrogel. We have previously demonstrated that DAPT-loaded MSNs can be used to inhibit Notch signaling *in vivo* and the technology was shown to be efficient both for active targeting of breast cancer stem cells as well as stem cell differentiation in the gastrointestinal tract when introduced intravenously and intraperitoneally in a sequential manner [22,50]. In the present study, even though our nanoparticle platform was not suitable for long-term sustained Notch inhibition, it was able to inhibit Notch transiently, showing promise for other biological and therapeutical applications where transient inhibition of Notch can prove beneficial. For instance, transient inhibition of Notch has been shown to promote osteoclastogenesis and remodeling during bone repair and enhance neuronal differentiation from neural stem cells [[87], [88], [89]]. It has also been suggested that the timely transient inhibition of Notch can be employed in controlling immune cell differentiation [90]. Thus, with the approach developed in this work, the site specificity of the DAPT-loaded particles can be further improved by their entrapment into a 3D matrix when modulating Notch while reducing the off-target effects of the Notch signaling inhibitor. Besides, further exploration on the choice of MSN size and pore size might be potentially impactful to modulate the drug release kinetics from nanocomposite hydrogels. Taken together, our platform can be used to deliver hydrophobic drug molecules in a wide range of therapeutical or tissue engineering contexts.

## 4 Conclusion

We have successfully demonstrated that surface modification is a critical parameter in the formulation of MSN-collagen nanocomposites, affecting cytocompatibility, cellular internalization, and hydrophobic cargo delivery in 3D. MSNs with negative (SUC) and neutral (ACA) net surface charge performed superior to obtain colloidally stable, homogenous MSN-collagen composites with good biocompatibility for myoblast cells among the other surface modifications investigated. PEI, ACA and SUC surface-modified MSNs were efficiently internalized by cells at different rates in a time-dependent manner, and nanoparticle-mediated drug delivery was achieved within the 3D matrices. Nanocomposite hydrogel incorporated with DAPT-loaded MSN-PEI-SUC could successfully downregulate Notch targeting genes and induce myogenesis, making this platform a promising candidate for steering cellular signaling in a complex environment. In conclusion, this proof-of-concept study opens up new possibilities for MSN-based 3D nanocomposites for tissue engineering, and a similar approach can be adapted to various tissue types, given that Notch signaling control stemness across a wide range of tissues. Additionally, it offers the flexibility that by tailoring the MSN pore size, drug molecules of different sizes can be incorporated; and furthermore, the resulting composite hydrogel can be utilized as a cell-laden biomaterial ink to achieve more intricate constructs with 3D bioprinting.

## CRediT authorship contribution statement

**Ezgi Özliseli:** Conceptualization, Methodology, Validation, Formal analysis, Investigation, Supervision, Visualization, Writing – original draft, Writing – review & editing. **Sami Şanlıdağ:** Investigation, Formal analysis, Writing – review & editing. **Behice Süren:** Investigation, Formal analysis. **Alaa Mahran:** Investigation, Formal analysis. **Marjaana Parikainen:** Formal analysis, Writing – review & editing. **Cecilia Sahlgren:** Conceptualization, Supervision, Resources, Funding acquisition, Writing – review & editing. **Jessica M. Rosenholm:** Conceptualization, Supervision, Resources, Funding acquisition, Project administration, Writing – review & editing.

## Declaration of competing interest

The authors declare that they have no known competing financial interests or personal relationships that could have appeared to influence the work reported in this paper.

## Data Availability

Data will be made available on request.
