# Supplementary material for: Directing cellular responses in a nanocomposite 3D matrix for tissue regeneration with nanoparticle-mediated drug delivery
Source: Mater Today Bio. 2023 Nov 14;23:100865. doi: 10.1016/j.mtbio.2023.100865 (PMC10694759; doi:10.1016/j.mtbio.2023.100865)
Supplement: Multimedia component 1 [file mmc1.docx]

**Supporting Information**

**Directing cellular responses in a nanocomposite 3D matrix for tissue regeneration with nanoparticle-mediated drug delivery**

*Ezgi Özliseli^1^,* *Sami Şanlıdağ^2,3,4^, Behice Süren^1^, Alaa Mahran^1,5^, Marjaana Parikainen^2,3,4^, Cecilia Sahlgren^2,3,4,6,7^*, Jessica M. Rosenholm^1^**

^1^Pharmaceutical Sciences Laboratory, Faculty of Science and Engineering, Åbo Akademi University, Turku, Finland

^2^Faculty of Science and Engineering, Biosciences, Åbo Akademi University, Turku, Finland

^3^InFLAMES Research Flagship Center, Åbo Akademi University, Turku, Finland

^4^Turku Bioscience Centre, Åbo Akademi University and University of Turku, Turku, Finland

^5^Department of Pharmaceutics, Faculty of Pharmacy, Assiut University, Assiut, 71526, Egypt

^6^Department of Biomedical Engineering, Eindhoven University of Technology, Eindhoven, the Netherlands

^7^Institute for Complex Molecular Systems (ICMS), Eindhoven University of Technology, Eindhoven, the Netherlands

*Correspondence: [jessica.rosenholm@abo.fi](mailto:jessica.rosenholm@abo.fi), [cecilia.sahlgren@abo,fi](mailto:cecilia.sahlgren@abo,fi)

**Table S1.** *Fluorescence intensity of MSNs*

|  | Fl intensity (a.u.) | Normalized value (a.u.) |
| --- | --- | --- |
| MSN | 925,5 | 2,33 |
| PEI | 461 | 1,16 |
| ACA | 2052,5 | 5,16 |
| SUC | 1414,5 | 3,56 |
| PEG | 397,5 | 1,00 |

| ­Primer | Forward sequence | Reverse sequence | NCBI ref. | Length (bp) |
| --- | --- | --- | --- | --- |
| Rpl13a | GTG GTC CCT GCT GCT CTC AAG | CGA TAG TGC ATC TTG GCC TTT T | NM_009438.5 | 152 |
| Hes1 | ACA CCG GAC AAA CCA AAG AC | AAT GCC GGG AGC TAT CTT TC | NM_008235.2 | 148 |
| Hey1 | CAC CTG AAA ATG CTG CAC AC | ATG CTC AGA TAA CGG GCA AC | NM_010423.2 | 122 |
| Myogenin | CCC AAC CCA GGA GAT CAT TTG | CAG TTG GGC ATG GTT TCG TC | NM_031189.2 | 134 |
| Mef2a | CAG GTG GTG GCA GTC TTG G | TGC TTA TCC TTT GGG CAT TCA A | NM_001357324.1 | 132 |
| Myh4 (MyH2B) | CAA TCA GGA ACC TTC GGA ACA C | GTC CTG GCC TCT GAG AGC AT | NM_010855.3 | 80 |

**Table S2.** Forward and reverse primer sequences used in qRT-PCR.


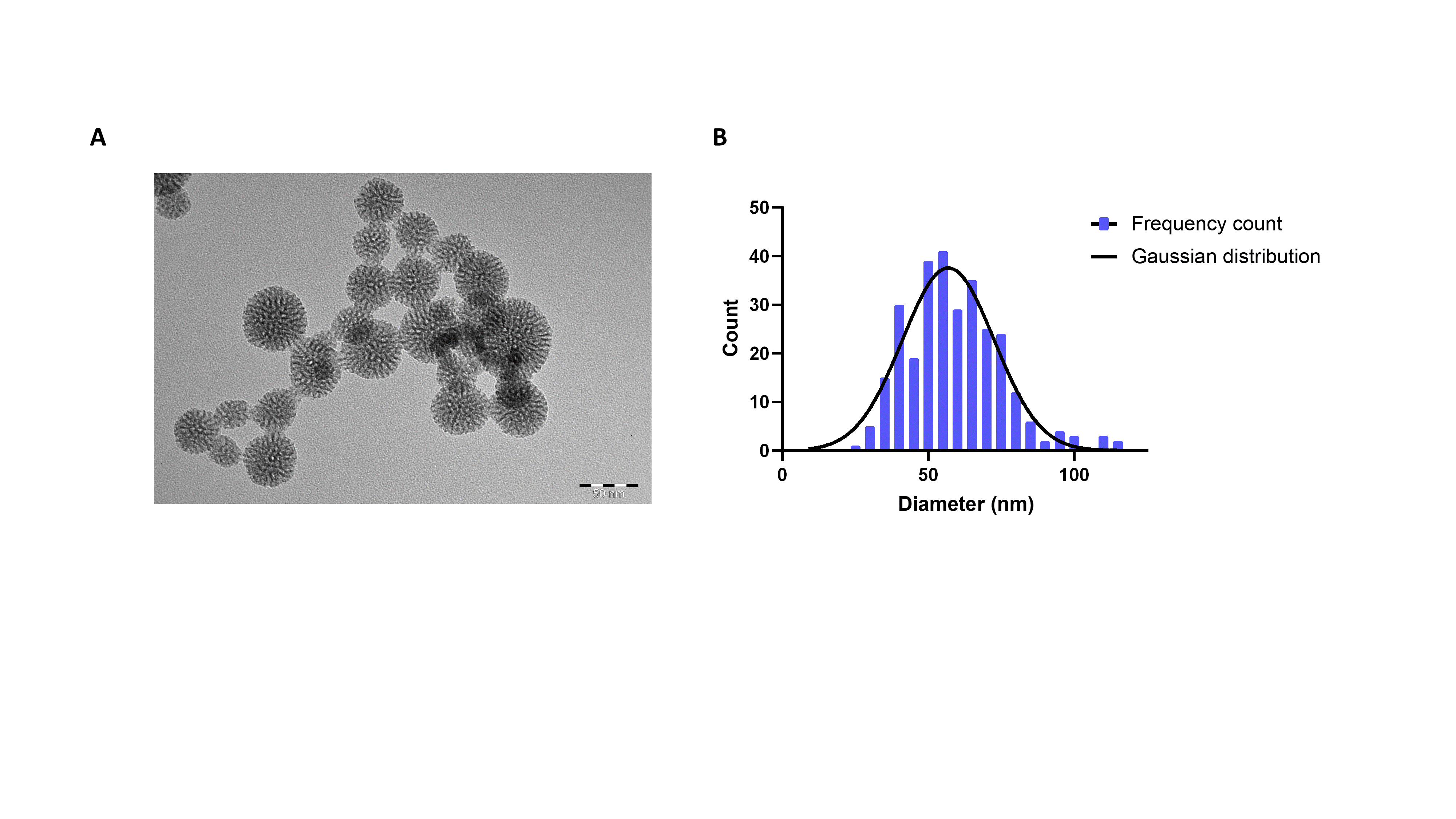


**Figure S1.** Transmission electron microscopy image analysis of mesoporous silica nanoparticles. A) Representative image acquired by TEM with scale bar 50 nm; B) Size distribution histogram of nanoparticles.


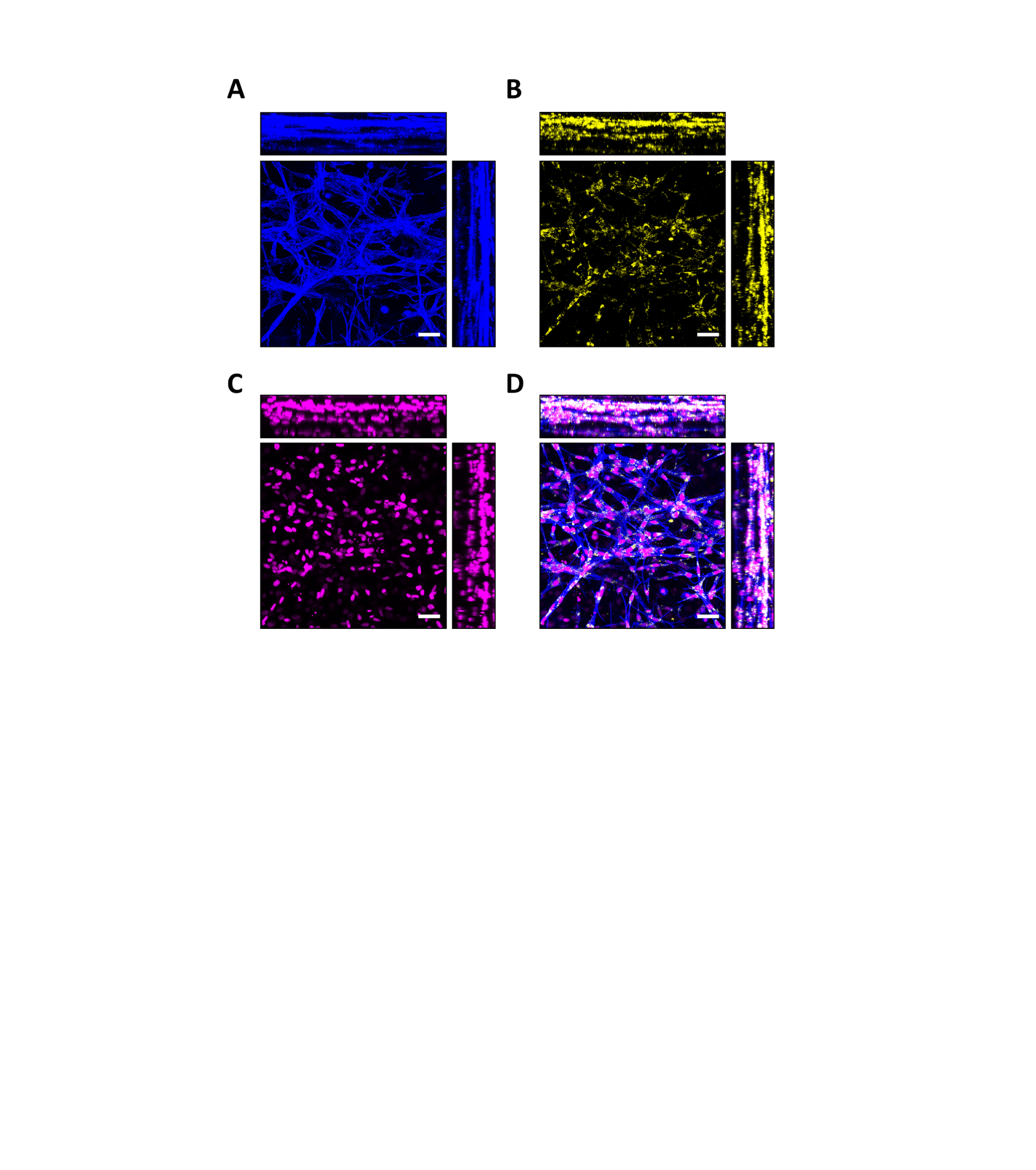


**Figure S2.** Orthogonal view of 3D composite hydrogel containing 50 µg/ml MSN-PEI-SUC nanoparticles after 24 h incubation exhibiting the spatial distribution of nanoparticle internalization within the cells **(A)** Phalloidin channel (blue) **(B)** TRITC channel (yellow) **(C)** DAPI channel (magenta) **(D)** merged channel of phalloidin, TRITC, DAPI, and internalized MSN (=colocalization of F-actin and TRITC-MSN, white). The scale bar corresponds to 50 µm.

**
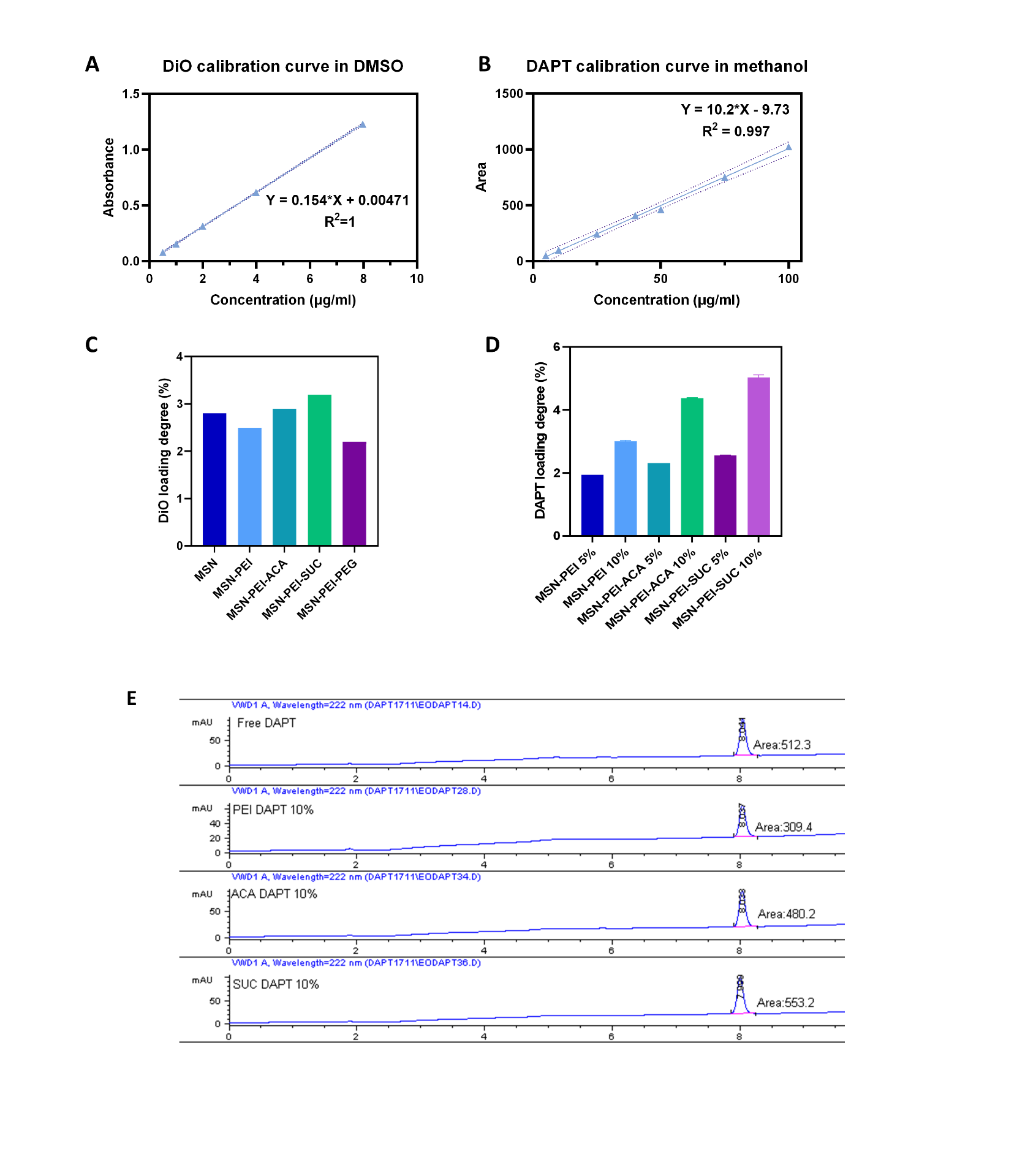
**

**Figure S3.** Hydrophobic cargo loading into the surface modified mesoporous silica nanoparticles. Calibration curve of DiO **(A)** and DAPT **(C)** for the calculation of loading degree in MSNs acquired by measuring absorbance in spectrophotometer for DiO and HPLC for DAPT. **(B)** Final wt% drug loading degree of DiO with the 5 % initial loading degree. **(D)** DAPT final wt% loading calculated with the 5 % or 10 % initial loading degree. (E) HPLC chromatograms of free DAPT and DAPT loaded MSNs with the theoretical loading degree of 10%. Number on the peak indicates the retention time and area is utilized to calculate the drug content in MSNs.


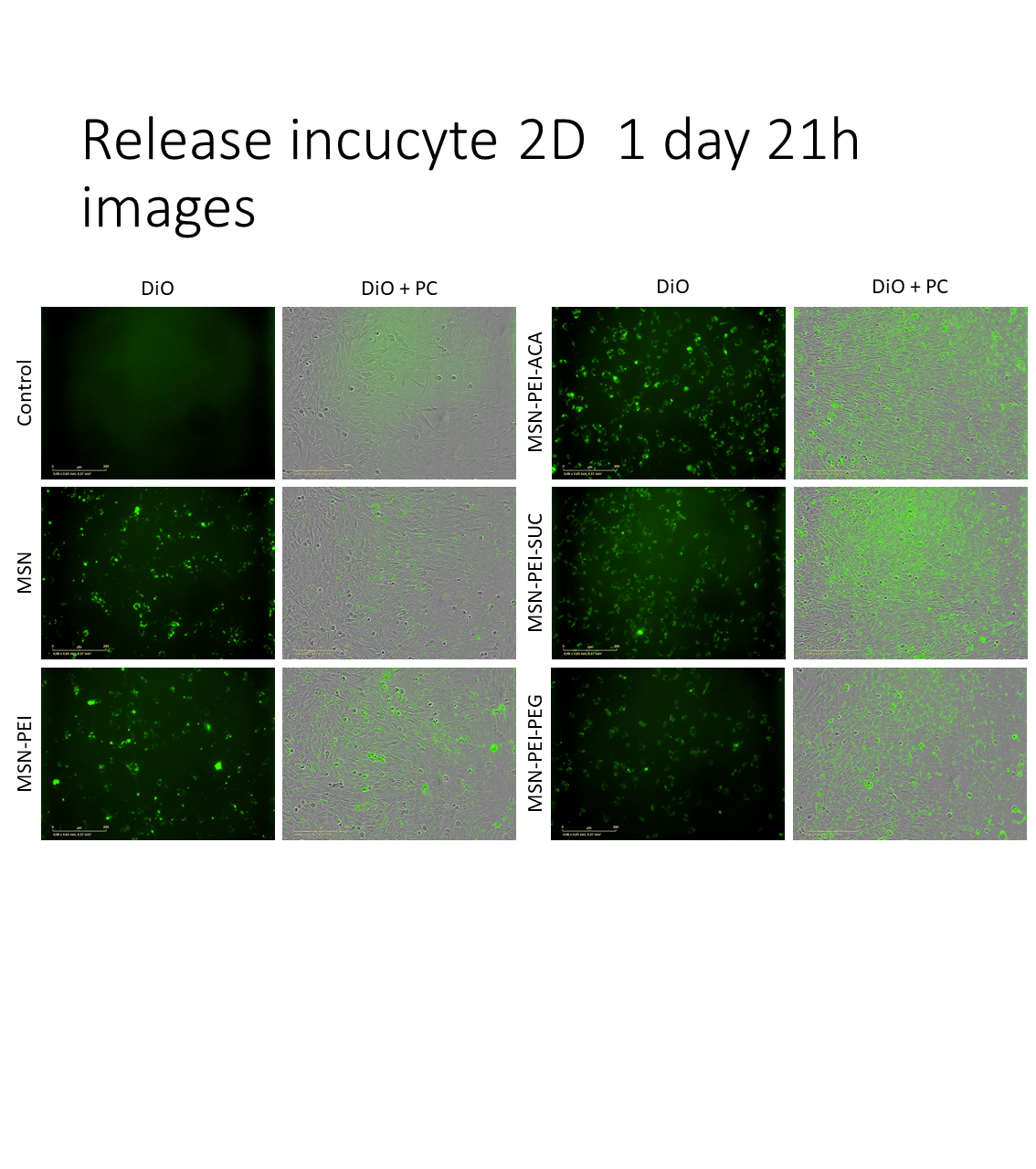


**Figure S4.** Green channel and phase contrast (PC) images acquired by IncuCyte from two different independent experiments after 48 h incubation with DiO loaded T-MSNs.


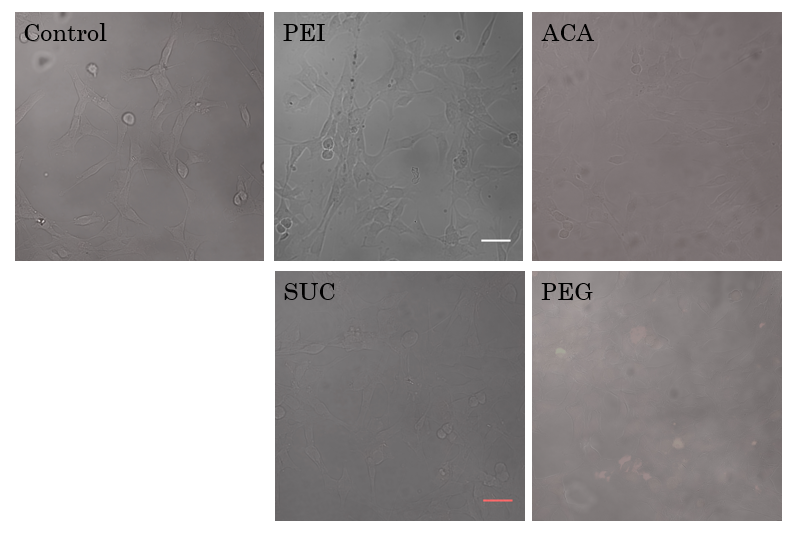
 **Figure S5.** Confocal microscopy images of cells after incubation of washing media for 48 h to verify escape of nanoparticles from the hydrogel. The images depict a composite of phase contrast, DiO, and TRITC-MSNs channels. Scale bar 50 µm.


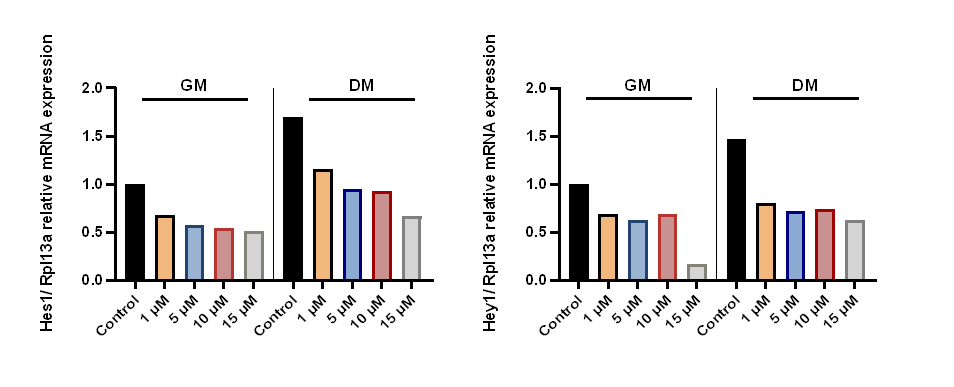
 **Figure S6.** Hes1 and Hey1 Notch target gene expressions determined by qPCR after DAPT treatment with various doses for 48 h in growth media (GM) or differentiation media (DM) supplemented with 1% horse serum.


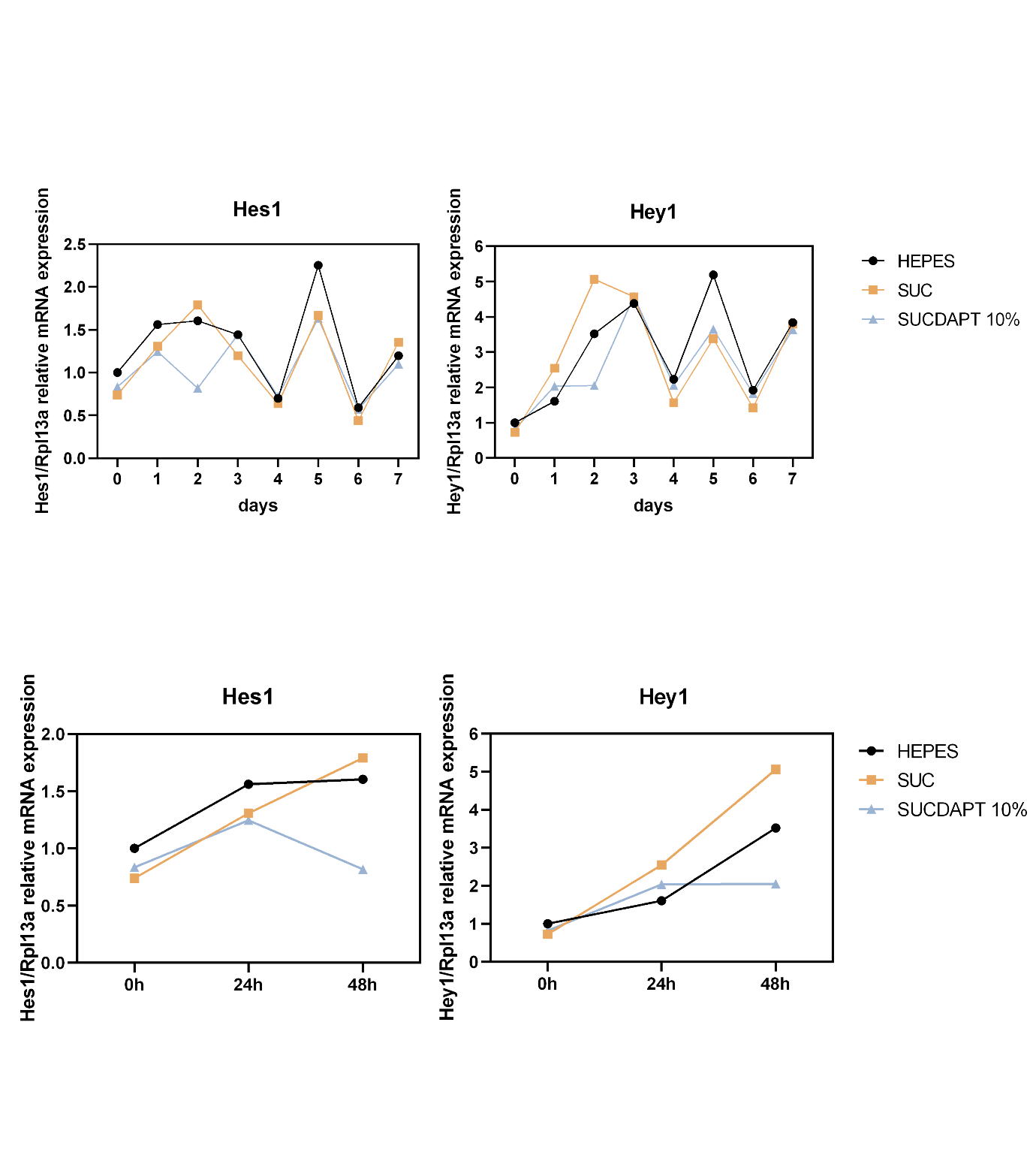
 **Figure S7.** Hes1 and Hey1 expressions of myoblasts in response to 5 µM DAPT containing MSN-PEI-SUC-DAPT treatment.


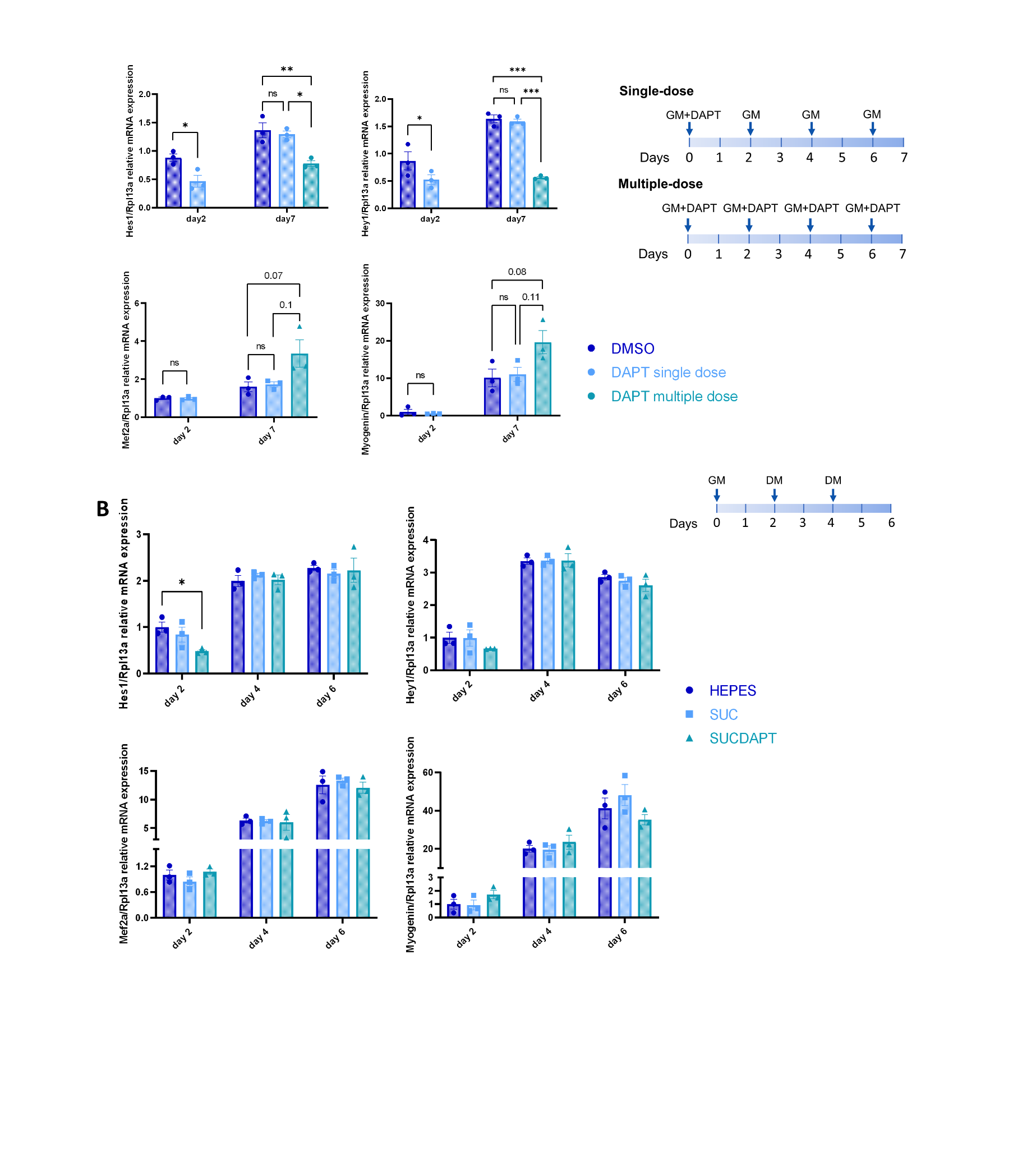


**Figure S8.** Expression of Notch signaling target genes and differentiation markers by the cells grown in 3D culture in response to single or repeated doses of free DAPT. Each experiment was carried out with three independent experiments (mean ± SEM, n=3). Two-tailed, unpaired student’s t-test was used to evaluate the significance between two groups, and one-way ANOVA with Tukey’s post-hoc was performed for comparison of more than two groups, *p < 0.05, **p < 0.01, ***p < 0.001.


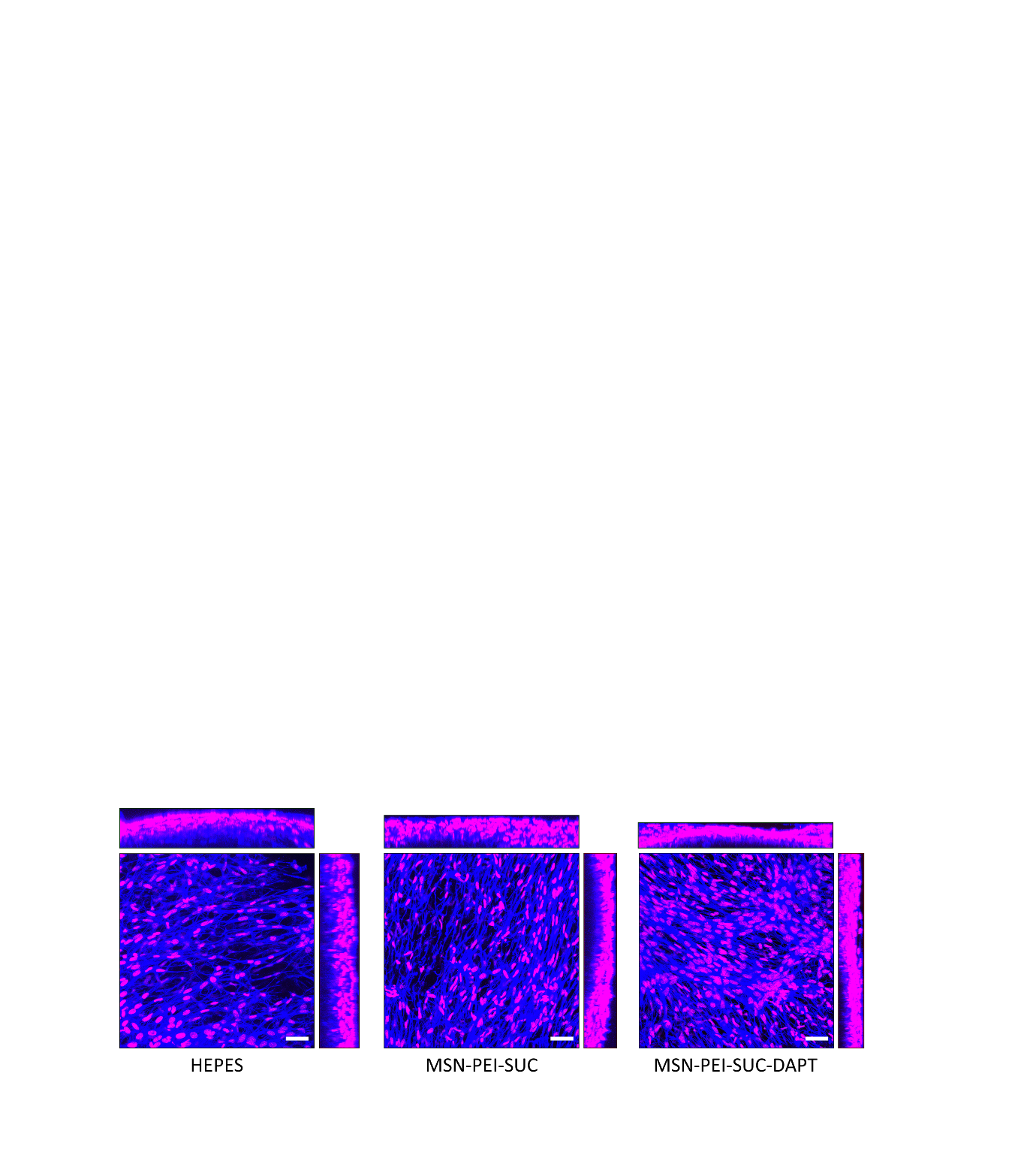


**Figure S9.** Orthogonal view of 3D composite hydrogels containing 5 µM DAPT-loaded MSN or empty MSN after 6 days of incubation exhibiting dense network, acquired by confocal microscopy. Images represent an overlay of F-actin staining with phalloidin (blue), and nuclei staining with DAPI (magenta). The scale bar is 50 µm.
