# Supplementary material for: Directing cellular responses in a nanocomposite 3D matrix for tissue regeneration with nanoparticle-mediated drug delivery
Source: Mater Today Bio. 2023 Nov 14;23:100865. doi: 10.1016/j.mtbio.2023.100865 (PMC10694759; doi:10.1016/j.mtbio.2023.100865)
Supplement: Multimedia component 2 [file mmc2.pptx]

## Slide 1
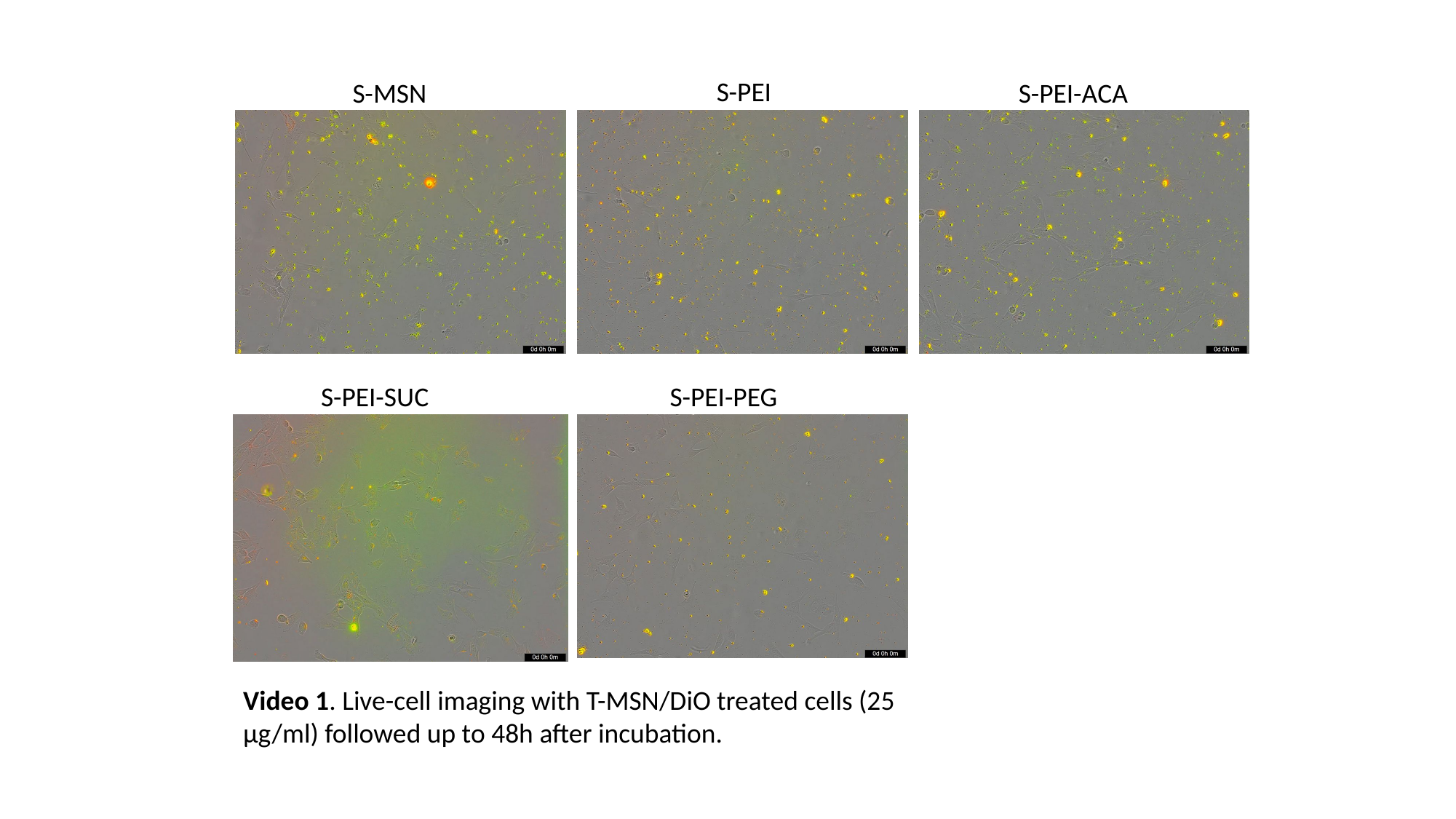

S-PEI
S-MSN
S-PEI-ACA
S-PEI-SUC
S-PEI-PEG
Video 1. Live-cell imaging with T-MSN/DiO treated cells (25 µg/ml) followed up to 48h after incubation.
